# The Histone Demethylase LSD1/ΚDM1A Mediates Chemoresistance in Breast Cancer via Regulation of a Stem Cell Program

**DOI:** 10.3390/cancers11101585

**Published:** 2019-10-17

**Authors:** John Verigos, Panagiotis Karakaidos, Dimitris Kordias, Alexandra Papoudou-Bai, Zoi Evangelou, Haralampos V. Harissis, Apostolos Klinakis, Angeliki Magklara

**Affiliations:** 1 Institute of Molecular Biology and Biotechnology-Foundation for Research and Technology, 45110 Ioannina, Greece; ioan_ver@yahoo.gr (J.V.); pkarakp@yahoo.gr (P.K.); d.kordias@hotmail.com (D.K.); 2 Department of Clinical Chemistry, Faculty of Medicine, University of Ioannina, 45110 Ioannina, Greece; 3 Biomedical Research Foundation Academy of Athens, 11527 Athens, Greece; aklinakis@bioacademy.gr; 4 Department of Pathology, University Hospital of Ioannina, 45500 Ioannina, Greece; apapoudoubai@gmail.com (A.P.-B.); zoe_evag@yahoo.com (Z.E.); 5 Department of Surgery, University Hospital of Ioannina, 45500 Ioannina, Greece; harissis.h@gmail.com

**Keywords:** breast cancer stem cells, tumor heterogeneity, therapy resistance, combination therapy, self-renewal, tumor initiation, lysine-specific demethylase 1, KDM1A

## Abstract

Breast cancer is the leading cause of cancer death in the female population, despite advances in diagnosis and treatment. The highly heterogeneous nature of the disease represents a major obstacle to successful therapy and results in a significant number of patients developing drug resistance and, eventually, suffering from tumor relapse. Cancer stem cells (CSCs) are a small subset of tumor cells characterized by self-renewal, increased tumor-initiation capacity, and resistance to conventional therapies. As such, they have been implicated in the etiology of tumor recurrence and have emerged as promising targets for the development of novel therapies. Here, we show that the histone demethylase lysine-specific demethylase 1 (LSD1) plays an important role in the chemoresistance of breast cancer cells. Our data, from a series of in vitro and in vivo assays, advocate for LSD1 being critical in maintaining a pool of tumor-initiating cells that may contribute to the development of drug resistance. Combinatory administration of LSD1 inhibitors and anti-cancer drugs is more efficacious than monotherapy alone in eliminating all tumor cells in a 3D spheroid system. In conclusion, we provide compelling evidence that LSD1 is a key regulator of breast cancer stemness and a potential target for the design of future combination therapies.

## 1. Introduction

Breast cancer is the most commonly occurring malignancy in women and, recently, it has become the leading cause of cancer-associated deaths worldwide, surpassing the fatality of lung cancer in the female population [1]. Breast tumors from different patients manifest significant diversity on the phenotypic and transcriptional levels, leading to their early classification into histological types and, to the more recent, identification of intrinsic molecular subtypes, respectively [2]. This information on inter-tumoral heterogeneity has had a major impact on the therapeutic decision-making process and it has greatly improved disease management and overall patient survival [3].

The advent of new technologies, based on next-generation sequencing, allows for a more comprehensive analysis of individual tumors, revealing the presence of distinct cancer cell subpopulations with different functional characteristics, including increased tumor-initiation capacity, therapy resistance, and metastatic ability [4]. This intra-tumoral heterogeneity, accounts largely for the observation that some breast cancer patients respond, initially, well to treatment, but later succumb to tumor relapse and metastasis [5]. This may be due to the presence of therapy-resistant cancer cells that are not sufficiently eliminated by conventional treatment schemes and drive tumor regrowth and development of metastatic lesions [6]. It is, therefore, of critical importance to delineate the mechanisms that underlie the malignant properties of these cells, and thereby pave the way for the development of novel, therapeutic modalities targeted against them.

The presence of such therapy-resistant cancer cell subpopulations can be attributed to various extrinsic or intrinsic factors, the latter including genetic and epigenetic variability [7]. Certain cellular clones may have accumulated beneficial genetic alterations during tumor evolution and may be better fit to survive after treatment. Other therapy-resistant clones may carry new mutations that arose under the pressure of treatment [8]. However, tumor heterogeneity may also stem from diversification of the epigenome, resulting in distinct epigenetic subclones that may exhibit different responses to therapy and variable tumor-initiating capacity [9,10,11,12]. Such pools of epigenetic variants that are resistant to therapy could limit the efficacy of conventional anti-cancer treatments and could become a source of tumor relapse. An early elegant study by Sharma et al. [13] had shown that drug-tolerant clones which displayed an altered chromatin profile could be eliminated by chromatin-modifying agents, providing a new window for therapeutic intervention. Consequently, taking into consideration epigenetic heterogeneity within tumors can further inform the design of new treatments against cancer [12].

Tumor subpopulations with resistance to common therapeutic regimens are often endowed with a stemness phenotype and are referred to as cancer stem cells (CSCs). A plethora of studies implicate these cells in the etiology of tumor recurrence [14,15,16], turning them into prime candidates for the development of specific therapies that could be applied in combination with conventional treatments that target the bulk of the tumor, aiming to achieve complete tumor eradication [17,18,19]. Multiple lines of evidence suggest that the epigenetic state of CSCs may shape their distinctive properties, including self-renewal, drug resistance, and tumor-initiation capacity [17,20,21]. As a result, therapeutic approaches, using epigenetic modulators against CSCs are currently being tested in clinical trials in various types of malignancy [21].

Lysine demethylase 1A (KDM1A/LSD1) is the first histone demethylase discovered and plays a central role in the regulation of pluripotency in embryonic stem cells and in the process of neurogenesis and hematopoiesis [22,23]. Altered LSD1 expression has emerged as a main feature of tumorigenesis, as the enzyme is reported to be upregulated in many different tumor types including several solid cancers and leukemias [24]. Several recent studies have also highlighted the role of LSD1 in cancer stemness [25,26]. The maintenance of a pool of functional leukemic stem cells in acute myeloid leukemia relies on LSD1 overexpression and the enzyme regulates an oncogenic program that supports tumor growth [27]. In glioblastoma [28] and hepatocellular carcinoma [29] stem cells, high LSD1 expression regulates their distinctive properties in vitro and in vivo; these are abolished upon LSD1 inhibition [28] or silencing [29]. In breast cancer, it has been shown that the deubiquitinase USP28 stabilizes LSD1 and regulates the self-renewal of breast CSCs (bCSCs) in vitro and tumor growth in vivo [30]. The histone H3K27 demethylase KDM6A (Lysine-specific demethylase 6A) forms a repressive complex along with LSD1 and other epigenetic regulators to silence epithelial-to-mesenchymal transition (EMT) -transcription factors and inhibit EMT-induced bCSC properties [31]. Along the same lines, a recent study established that knock-down of LSD1 nearly abolished the CSC subpopulation after EMT induction in MCF-7 cells [32].

In the present study, we provide new, compelling evidence that corroborate LSD1’s involvement in the chemoresistance of breast cancer cells. By pharmacologically inhibiting LSD1 and genetically manipulating its expression, we show that it plays an important role in doxorubicin resistance by maintaining a pool of functional bCSCs. We found that LSD1 is an important regulator of self-renewal of bCSCs in vitro, in accordance with the aforementioned studies. However, we extend these findings further by presenting results from breast cancer patient samples, as well as in vivo data generated from limiting dilution assays in mice that revealed a dramatic decrease in tumor-initiating capacity, when LSD1 was inhibited or knocked-down, respectively. We also show that, upon pharmacological inhibition of LSD1 in mouse xenografts, the tumor CSC subpopulation was diminished, strengthening further the argument that this histone demethylase constitutes an essential molecular component of the stemness phenotype in breast cancer. Finally, we show that combination treatment including an LSD1 inhibitor and a cancer drug is more efficacious in eliminating all neoplastic cells in 3D tumorsphere models of breast cancer. Such chemotherapeutic schemes may hold promise for better clinical results in the oncological practice in the future.

## 2. Results

### 2.1. LSD1 Expression Is Associated with Aggressive and Poorly Differentiated Breast Carcinomas

An older study reported that LSD1 was overexpressed in estrogen receptor (ER) positive breast tumors and was a predictor of aggressive disease [33]. Subsequent studies showed that LSD1 levels were elevated during tumor progression of ductal carcinoma [34] and established that high LSD1 levels correlated with poor prognosis in breast cancer patients [35,36].

To confirm that *LSD1* is overexpressed in aggressive breast tumors, we searched gene expression data from relevant clinical samples using Oncomine [37] and the results are presented in Supplementary Materials Figure S1. The *LSD1* mRNA levels were significantly increased in specimens from patients with invasive breast cancer compared to normal breast tissue samples [38] (Figure S1A). These finding were corroborated by a second study [39], which provided gene expression data per breast tumor type (Figure S1B). Lysine-specific demethylase 1 was significantly upregulated both in invasive ductal and invasive lobular breast carcinomas, compared to normal breast samples (Figure S1B). In two other datasets [40,41], we chose to examine *LSD1* expression per tumor grade and the results are shown in Figure S1C,D. Higher *LSD1* expression levels were noted in poorly differentiated, grade 3 tumors.

Collectively, all the above clinical studies confirm that LSD1 is upregulated in aggressive breast cancers with poor prognosis, building a case that supports its involvement in the particularly malignant characteristics of these tumors.

### 2.2. LSD1 Mediates Resistance to Doxorubicin in Breast Cancer Cells

Given the association of LSD1 expression with more aggressive types of breast cancer that tend, frequently, to respond poorly to standard treatment and develop therapy resistance, we reasoned that LSD1 might play a role in rendering neoplastic cells less sensitive to drugs.

**Figure 1:**
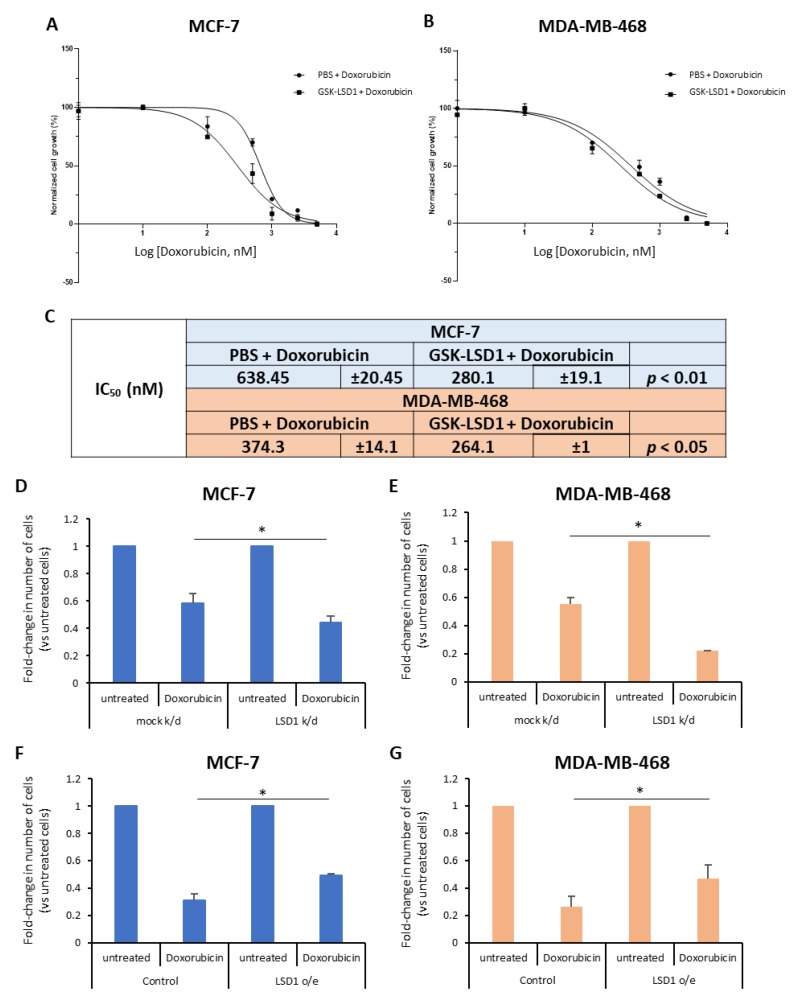
Lysine-specific demethylase 1 (LSD1) mediates doxorubicin resistance in breast cancer cells. (**A**) MCF-7 and (**B**) MDA-MB-468 breast cancer cells were treated with vehicle (phosphate-buffered saline, PBS) or GSK-LSD1 inhibitor (0.5 μM) for 5 days before the addition of increasing concentrations (0–5 μΜ) of doxorubicin for two more days. Cell confluency was measured using the Incucyte Zoom live cell analysis system. (**C**) The doxorubicin IC_50_ values in MCF-7 and MDA-MB-468 cells with or without pretreatment with the inhibitor GSK-LSD1. IC_50_ calculation was performed using Graphpad Prism version 8.01. Data from two independent experiments performed in triplicate are shown. (**D**) MCF-7 and (**E**) MDA-MB-468 breast cancer cells were knocked-down with an siRNA for LSD1. Four days post-transfection, cells were treated with doxorubicin for 24 h, and the number of live cells was counted. Mock knock-down was performed using a scrambled siRNA. (**F**) MCF-7 and (**G**) MDA-MB-468 breast cancer cells were transfected with an empty (control) or an LSD1 expression vector. Forty-eight hours post-transfection, cells were treated with doxorubicin for 24 h, and the number of live cells was counted. Error bars represent SEM. * *p* < 0.05.

To this end, we treated ΜCF-7 and MDA-MB-468 breast cancer cells with a highly specific LSD1 inhibitor, GSK-LSD1 [42] or vehicle (phosphate-buffered saline, PBS) for 7 days and, also, exposed them to increasing doses of doxorubicin (0–5 μM), a drug commonly given to breast cancer patients, for the last 2 days. The effects on cell proliferation were monitored using real-time imaging with the Incucyte ZOOM system. Our data showed that doxorubicin treatment alone resulted in considerable decrease of cell growth in both cell lines (Figure 1A,B), as expected. Remarkably, pre-treatment with the LSD1 inhibitor significantly enhanced the drug’s effects on cell proliferation (Figure 1A,B). Specifically, upon pre-treatment with GSK-LSD1, the IC_50_ values for doxorubicin decreased significantly from 0.64 μΜ and 0.37 μM to 0.28 μΜ and 0.26 μM in MCF-7 and MDA-MB-468 cells, respectively (Figure 1C). These results suggest that LSD1 confers doxorubicin resistance to breast cancer cells.

To further support the above data, we performed knock-down of *LSD1*, in these two cell lines, using a previously validated siRNA [43] specific for the enzyme. Western blot analysis confirmed a highly efficient knock-down of protein expression in both MCF-7 (Figure S2A) and MDA-MB-468 (Figure S2B) cells. Four days post-transfection, the cells were treated with 2.5 μΜ (for MCF-7) or 1 μΜ (for MDA-MB-468) doxorubicin for 24 h and, subsequently, the number of live cells was recorded (Figure 1D,E). Reduction of LSD1 levels rendered the cells more sensitive to chemotherapy, as indicated by the significant decrease in the number of cells that survived after drug treatment. Approximately, 15% fewer LSD1-deficient MCF-7 (Figure 1D) and 30% fewer LSD1-defiecient MDA-MB-468 cells (Figure 1E) survived after doxorubicin treatment, compared to scrambled siRNA-transfected cells, providing additional evidence that LSD1 plays a role in the chemoresistance of breast cancer cells.

In complementary experiments, cells were transfected with an expression vector for LSD1. Forty-eight hours later, they were treated with 2.5 μΜ (MCF-7) or 1 μΜ (MDA-MB-468) doxorubicin for 24 h and the live cells were counted at the end of the experiment. Cells were harvested and LSD1 protein levels were analyzed by Western blot to confirm LSD1 overexpression (Figure S2C,D). LSD1 overexpression conferred resistance both to MCF-7 (Figure 1F) and MDA-MB-468 cells (Figure 1G) against doxorubicin-induced death, leading to a significant increase in the percentage of cancer cells that survived after drug treatment. Approximately, 20% more LSD1-overexpressing cells survived, compared to mock-transfected cells, in both cell lines (Figure 1F,G).

Taken together, the above data suggest that LSD1-regulated mechanisms may contribute to the resistance that, oftentimes, breast cancer cells develop against therapeutic agents, such as doxorubicin, commonly used in the oncological practice.

### 2.3. LSD1 Regulates the Stemness Properties of Breast Cancer Stem Cells In Vitro

According to the literature [33,34,35,36] and the studies described before (Section 2.1), LSD1 is overexpressed in aggressive and poorly differentiated breast tumors that are known to be enriched in CSCs [44]. Furthermore, based on our data presented above, the histone demethylase mediates drug resistance in breast cancer cells, an important property of CSCs [45]. Therefore, we set out to investigate whether LSD1 confers chemoresistance via regulation of the bCSC subpopulation.

Previous work had shown that doxorubicin-resistant MCF-7 cells were enriched in CSCs compared to the parental cell line [46]. We sought to confirm that our MCF-7 and MDA-MB-468 cell lines contained CSCs that preferentially survived chemotherapy with common breast cancer drugs, such as doxorubicin and paclitaxel. To this end, both cell lines were treated with different concentrations of doxorubicin or paclitaxel (described in Materials and Methods) for 2 or 6 days, respectively, and the number of live cells was recorded on the first and last day of treatment. Representative data are presented in Figure S3A. As expected, most cells died after drug treatment. The remaining live cells were subjected to FACS analysis and the CSC subpopulation was monitored using the well-characterized breast CSC-phenotype CD44^+^/CD24^-/low^ [47]. Our results revealed that the cells that survived drug treatment were significantly enriched in CSCs (Figure S3B), verifying that this subpopulation had increased resistance to the two drugs. Remarkably, the CSC subpopulation was enriched in the MCF-7 cells by −60% after doxorubicin and by 4% after paclitaxel treatment (Figure S3B, blue panel). The results were less profound but still consistent in the MDA-MB-468 cells, where doxorubicin administration induced a −5% and paclitaxel a 3% enrichment in CSCs compared to control untreated cells (Figure S3B, red panel). Thus, these data confirmed the presence of drug resistant stem-like cells in both cell lines used, therefore, validating their choice for subsequent experiments.

**Figure 2:**
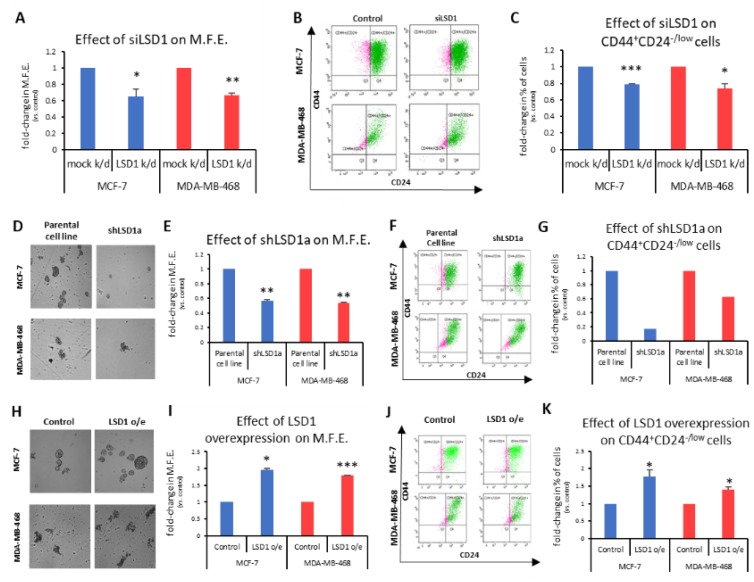
LSD1 regulates the stemness properties of bCSCs. (**A**) Graphical representation of the effects of LSD1 knock-down on the mammosphere forming efficiency (MFE) MCF-7 and MDA-MB-468 cells were transfected with siRNA against *LSD1* and cultured under mammosphere-forming conditions for 7 days when the MFE was calculated. Cells transfected with scrambled siRNA served as the control. (**B**) FACS analysis for CD44^+^CD24^-/low^ cells in MCF-7 and MDA-MB-468 mammospheres after siRNA-mediated LSD1 knock-down. (**C**) Quantification of FACS analysis presented in (**C**). (**D**) Representative images of MCF-7 and MDA-MB-468 mammospheres derived from parental and stable LSD1 knock-down cells after 7 days in culture. (**E**) Graphical representation of the effects of stable LSD1 knock-down on the MFE of MCF-7 and MDA-MB-468 mammospheres after 7 days in culture under mammosphere-forming conditions. Parental cell lines served as control. (**F**) FACS analysis for CD44^+^CD24^-/low^ cells in MCF-7 and MDA-MB 468 mammospheres derived from parental and stable LSD1 knock-down cell lines. (**G**) Quantification of FACS analysis presented in (**F**). Data from one experiment performed in triplicate, pooled for FACS analysis, are shown. (**H**) Representative images of MCF-7 and MDA-MB-468 mammospheres derived from control and LSD1-overexpressing cells after 7 days in culture. (**I**) Graphic representation of the effects of LSD1 overexpression on the MFE of MCF-7 and MDA-MB-468 mammospheres. Cells transfected with empty vector served as control. (**J**) FACS analysis for CD44^+^CD24^-/low^ cells in MCF-7 and MDA-MB-468 mammospheres after LSD1 overexpression. (**K**) Quantification of FACS analysis presented in (**I**). Error bars represent SEM. * *p* < 0.05, ** *p* < 0.01, *** *p* < 0.001.

To investigate the role of LSD1 in the regulation of bCSCs, we employed the mammosphere-forming assay, an in vitro surrogate tool to evaluate stemness [48]. First, a specific siRNA [43] was used to transiently knock-down *LSD1* gene expression in MCF-7 and MDA-MB-468 cells. Western blot analysis demonstrated that reduced LSD1 levels persisted 7 days post-transfection (Figure S4A), which was the duration of the mammosphere-forming experiments. These experiments exhibited a significant decrease in the ability of knocked-down cells to form mammospheres in both cell lines tested (Figure 2A). Specifically, the transiently LSD1-knock-down cells were −35% less efficient in forming spheres than the control cells transfected with scrambled siRNA (Figure 2A). The last day of the experiment, the mammospheres were collected and dissociated to single cells, which were, subsequently, subjected to FACS analysis to monitor the CD44^+^CD24^-/low^ CSC subpopulation (Figure 2B). Analogous to the mammosphere forming efficiency (MFE), a significant reduction of the CSC subpopulation was observed, ranging from 21% to 26%, in the MCF-7 and MDA-MB-468 cells, respectively (Figure 2C).

To confirm the above data, we also used two different shRNAs against *LSD1* (shLSD1a and shLSD1b, Figure S4B) in MCF-7 and MDA-MB-468 cells and generated three stable knock-down cell lines (MCF-7_shLSD1a, MCF-7_shLSD1b, and MDA-MB-468_shLSD1a), as we could not obtain a stable clone with shLSD1b in the latter cell line. Western blot analysis confirmed that LSD1 was depleted in all three stable clones (Figure S4C).

These three stable cell lines were used in subsequent experiments to examine the effects of LSD1 depletion on the CSC subpopulation and the results are presented in Figure 2D–G and Figure S4D,F. The MCF-7_shLSD1a and MDA-MB-468_shLSD1a cells were cultured under mammosphere-forming conditions for 7 days to examine their ability to form spheres (Figure 2D and enlarged images provided in Supplementary Figure S4F). The LSD1 depletion led to a significant reduction in the MFE, as both knock-down cell lines were −45% less efficient in forming spheres compared to the parental ones (Figure 2E). This effect was even more prominent with the MCF-7_shLSD1b stable cell line, where the cells were −60% less capable in forming spheres compared to the parental cells (Figure S4D). The reduction in the MFE was accompanied by a decline in the CD44^+^CD24^-/low^ subpopulation, as monitored by FACS analysis, in the respective cell lines. Notably, both of the stable LSD1-deficient MCF-7 cell lines exhibited a dramatic decrease in CSCs, with the population dropping by 83% and 70% in the MCF-7_shLSD1a (Figure 2G, blue panel) and MCF-7_shLSD1b cells (Figure S4E), respectively. The pool of CSCs was also reduced in the MDA-MB-468_shLSD1a cells by 47% (Figure 2G, red panel). Taken together, the above data demonstrate that cells lacking in *LSD1* expression exhibit a significant loss of stemness potential, associated with a drop in the number of CSCs, as indicated by the reduction in their MFE and in the CD44^+^CD24^-/low^ subpopulation, respectively.

In complementary experiments, we transfected MCF-7 and MDA-MB-468 cells with an expression vector for *LSD1*. Increased LSD1 protein levels were confirmed by Western blot analysis (Figure S4G). The MCF-7- and MDA-MB-468-mock-transfected and -LSD1-overexressing cells were cultured under mammosphere-forming conditions for 7 days, to examine their ability to form spheres (Figure 2H and enlarged images provided in Figure S4H). Upon LSD1 overexpression, there was a significant enhancement in the stemness potential of both cell lines, as this was demonstrated by an increase in their mammosphere-forming capacity (Figure 2I). The FACS analysis (Figure 2J) confirmed a concomitant small but significant growth of the CD44^+^CD24^-/low^ CSC subpopulation in both cell lines (Figure 2K).

Altogether, the above data strongly suggest that LSD1 is implicated in the regulation of breast cancer stemness properties and it is required for the maintenance of the CSC pool in vitro.

### 2.4. LSD1 Regulates Tumor-Initiation in MDA-MB-468 Mouse Xenografts In Vivo

In light of the above data—which showed a well-defined reduction of the mammosphere-forming efficiency of LSD1-deficient bCSCs in vitro—we sought to investigate whether LSD1 also impacts their tumor-forming ability in vivo. To this end, the limiting dilution assay (LDA), which is considered the gold standard to gauge tumorigenicity and frequency of functional CSCs [49], was employed. Equal numbers (5 × 10^6^, 1 × 10^6^, 1 × 10^5^) of parental or MDA-MB-468-shLSD1a cells were orthotopically transplanted in one mammary fat pad per mouse (*n* = 5) and monitored for 14 or 18 weeks, respectively. The parental cells formed tumors successfully at the two lowest dilutions (100%, 5/5) and with a slightly lower incidence at the third dilution (80%, 4/5 mice) (Table 1). A higher dilution (1 × 10^4^) of parental cells was also used which did not yield any tumors during the course of the experiment. Representative images from all groups of mice used in this experiment are shown in Figure S5.

The frequency of CSCs was calculated using the extreme limited dilution analysis (ELDA) [50], and it was estimated at −1/77000 cells in the MDA-MB-468 cells. Notably, stable LSD1 knock-down MDA-MB-468 cells were unable to induce tumor formation during the given time period, even at the higher inoculation numbers. We cannot exclude the possibility though that tumors might have formed within a longer time frame. These results suggest that LSD1 silencing strongly inhibits tumor initiation in vivo, a key functional property of CSCs.

### 2.5. LSD1 Expression Is Associated with Expression of the Stem Cell Marker CD44 in High-Grade Triple-Negative Breast Cancers 

In order to confirm the association of LSD1 with breast cancer stem cells, we performed immunohistochemistry for LSD1 and CD44 in a small set (*n* = 10) of tissue sections from patients with invasive triple-negative breast cancer (TNBC). The cell surface marker CD44 has been associated with stem-cell-like characteristics [51] and poor clinical outcome in breast cancer [52,53]. Taking into account that most breast tumors contain a small sub-population of bCSCs, we selected a set of high-grade TN tumors (9/10 were Grade 3 and 1/10 was Grade 2) that were expected to be highly enriched in CSCs [44,54,55].

**Figure 3:**
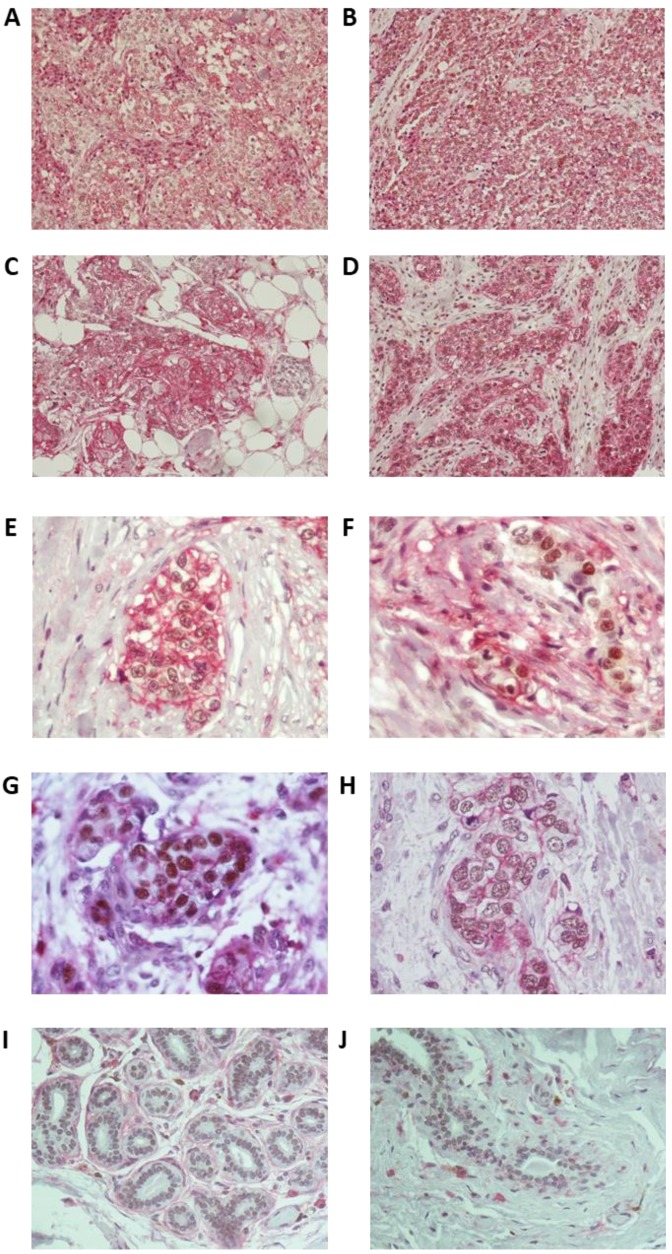
Immunohistochemistry in breast tumor sections from triple-negative breast cancer (TNBC) patients for LSD1 and the stemness marker CD44. (**A**–**D**) Representative images from four different tumors (cases 4, 8, 9, and 10 in Table S1) showing high enrichment in CD44^+^ cells and high co-expression of CD44 and LSD1. Magnification 200×. (**E**) Very high (−100%) and (**F**) lower (−40%) concomitant expression of LSD1 and CD44 in neoplastic cells. (**G**) Strong and (**H**) moderate nuclear staining for LSD1 in neoplastic cells. Magnification 600×. (**I**,**J**) Non-neoplastic cells of the adjacent breast parenchyma with weak nuclear LSD1 staining from two different tumors (magnification 400×). The cell surface antigen CD44 exhibits membranous staining (chromogenic substrate: magenta) and LSD1 is nuclear (chromogenic substrate: DAB).

In 8 out of 10 cases examined, the vast majority of cancer cells (80–100%) were CD44^+^ (Table S1) indicating that these tumors were, indeed, highly enriched in bCSCs. Representative pictures of tumor sections are shown in Figure 3A–D. Interestingly, all the CD44-positive cancer cells were also LSD1 positive, strongly suggesting an association of CSCs with LSD1 expression. The lowest levels of co-expression (40%) were found in the grade 2 tumor that also had the lowest content in CD44^+^ cells (Table S1). Overall, the concomitant staining of LSD1 (nuclear) and CD44 (membranous) ranged from 40% to 100%, with most of the tumors showing >80% co-expression (Figure 3A–D). The variable co-expression of LSD1 and CD44 is shown in Figure 3E,F. Co-expression of the two proteins is very high (−100%) in the tumor section shown in Figure 3E and lower (−40%) in the section shown in Figure 3F.

Regarding the levels of LSD1 expression, most of the neoplastic breast epithelial cells in six tumors expressed LSD1 with moderate to strong intensity, while in four tumors, the high expressing LSD1 cancer cells ranged from 20–50%. Representative images of the different levels of LSD1 expression in the tumor sections are presented in Figure 3G (strong) and Figure 3H (moderate). Remarkably, the majority of the adjacent non-neoplastic epithelial cells also expressed LSD1, albeit with weak intensity, confirming that LSD1 is upregulated in cancer (Table S1). Non-neoplastic tissue with weak LSD1 staining from two different specimens is shown in Figure 3I,J.

Despite the small number of clinical specimens examined, these data confirm that poorly differentiated tumors enriched in CSCs express high levels of LSD1. It is, therefore, plausible that LSD1 is important for the maintenance of the bCSC pool, as our previous results had indicated.

### 2.6. LSD1 Pharmacological Inhibition Affects CSC Self-Renewal In Vitro and In Vivo

The above data confirm that LSD1 plays a critical role in regulating the fundamental properties of bCSCs in vivo and in vitro; consequently, its pharmacological targeting could, potentially, lead to a significant reduction or even elimination of these cells.

**Figure 4:**
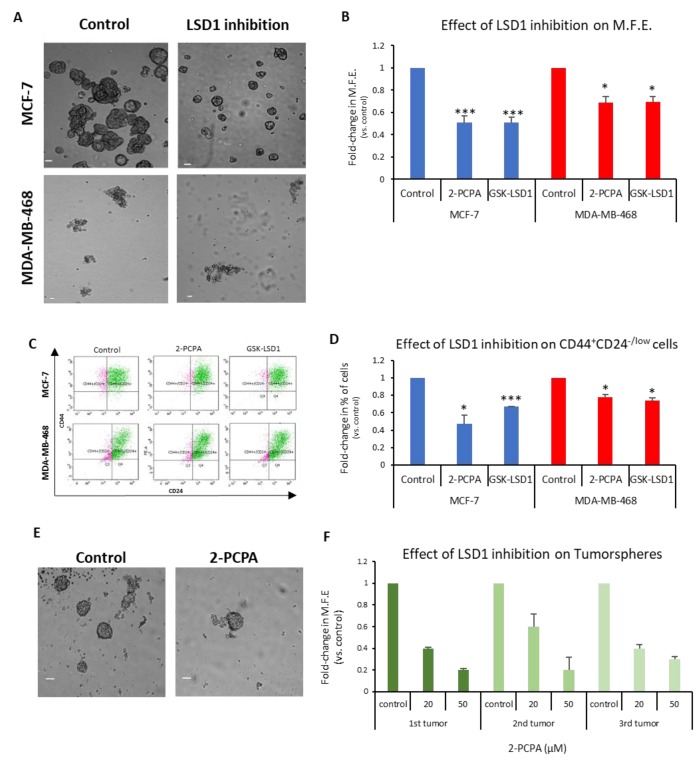
Pharmacological inhibition of LSD1 affects the stemness properties of bCSCs. (**A**) Representative images of MCF-7 and MDA-MB-468 mammospheres after inhibition of LSD1 with 2-PCPA (50 μΜ) for 7 days. Vehicle-treated spheres served as the control. (**B**) Graphical representation of the effects of LSD1 inhibition on the MFE MCF-7 and MDA-MB-468 mammosphere-derived single cells. The cells were treated with 2-PCPA (50 μΜ) or GSK-LSD1 (2 μΜ) for 7 days and, on the last day, the number of formed mammospheres was counted and the MFE was calculated. (**C**) FACS analysis for the CD44 and CD24 cell surface markers was performed for the mammosphere-derived single cells after inhibitor treatment. Vehicle-treated cells were used as control. (**D**) Quantification of FACS analysis data presented in (**C**). (**E**) Representative images of patient tumorspheres after treatment with vehicle (control) or 2-PCPA (50 μΜ) for 7 days. (**F**) Graphical representation of the effects of LSD1 inhibition on the MFE of patient tumorspheres. Patient tumorsphere-derived single cells were cultured, under mammosphere-forming conditions, in the presence of 2-PCPA (20 or 50 μΜ) for 7 days. On the last day of treatment, the number of tumorspheres was counted and the MFE was calculated. Error bars represent SEM. * *p* < 0.05, *** *p* < 0.001. In (**F**), error bars represent the SEM of technical replicates (*n* = 3–4). Scale bars represent 50 μm.

To investigate this, we used the two LSD1 inhibitors 2-PCPA and GSK-LSD1, as described in the Materials and Methods section, and representative data are shown in Figure 4A–D.

The MCF-7 and MDA-MB-468 first-generation mammospheres were dissociated to single cells, re-plated under mammosphere-forming conditions, treated with the two inhibitors for 7 days (Figure 4A), and the MFE was calculated (Figure 4B). The LSD1 inhibition resulted in a significant decline, approximately 50%, in the number of MCF-7 mammospheres (Figure 4B, blue panel). Both inhibitors also caused a significant reduction in the MFE of MDA-MB-468 spheres, but to a lesser extent (Figure 4B, red panel). The FACS analysis of these mammospheres (Figure 4C) revealed a significant decrease in the CD44^+^CD24^-/low^ subpopulation compared to vehicle-treated mammospheres in both cell lines (Figure 4D). Administration of 2-PCPA had a more severe effect on the CSC pool in the MCF-7 mammospheres, where it resulted in a 53% reduction of the population, whereas GSK-LSD1 had a milder effect causing a 33% reduction (Figure 4D, blue panel). The two inhibitors had a significant, albeit weaker (22–26%), effect on the number of CSCs in the MDA-MB-468 mammospheres, as well (Figure 4D, red panel). These results strongly suggest that LSD1 inhibition diminishes the stemness potential of CSCs and directly targets the CSC subpopulation in breast cancer. It is also evident that LSD1 pharmacological inhibition phenocopies the effects of the knock-down, as described previously (Figure 2) and, therefore, may serve as a therapeutic avenue for targeting this molecule and associated stemness pathways in breast CSCs.

In this direction, we examined the effects of LSD1 inhibition on tumorspheres derived from breast cancer patient tumor samples. Clinical specimens from 3 patients undergoing therapeutic mastectomy were dissociated and cells were grown under mammosphere-forming conditions for 7–10 days until tumorspheres (≥50 μΜ) were formed. Subsequently, the tumorspheres were collected, dissociated to single cells, re-plated under mammosphere-forming conditions, and treated with vehicle (Figure 4E, upper image) or 2-PCPA (20 or 50 μM) (Figure 4E, lower image) for 7 days, when the MFE was calculated (Figure 4F). Vehicle-treated tumorspheres could generate new spheres efficiently, confirming that they contained cells with self-renewal properties. The LSD1 inhibition resulted in a dose-dependent reduction in the tumorsphere-forming efficiency, suggesting that it, negatively affected the stemness potential of the CSC subpopulation present in breast cancer specimens. These results further support our in vitro data with the cell lines and underscore the importance of LSD1 inhibition in the clinical setting.

Next, we sought to investigate in vivo the employment of LSD1 pharmacological inhibition as a means to abolish bCSCs. To this end, we generated orthotopic mouse xenografts using MDA-MB-468 cells, as described in the Materials and Methods section. After inoculation of the cells and tumor formation, mice were administered 1 mg/kg of GKS-LSD1 inhibitor (*n* = 6) or vehicle (*n* = 5) for 3 weeks.

At the end of this time period, the mice were sacrificed and the excised tumors were measured, weighted, and processed for FACS analysis.

**Figure 5:**
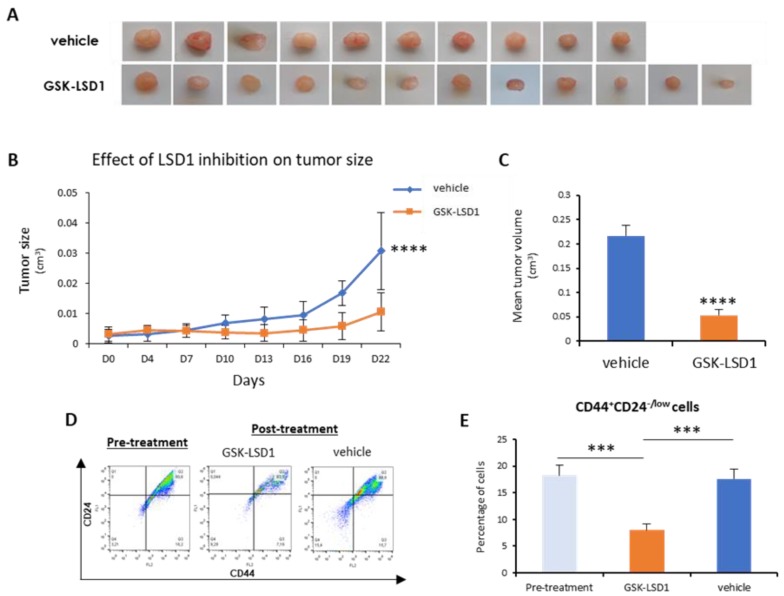
LSD1 inhibition reduced tumor growth and targeted the CSC subpopulation in vivo. MDA-MB 468 breast cancer cells were injected orthotopically in mice (into two fat pads/mouse). Treatment with the GSK-LSD1 inhibitor (*n* = 6) or with the vehicle control (*n* = 5) started when the tumors were detectable by palpation and lasted for 22 days, when the tumors were removed and measured. (**A**) Representative images of the tumors excised from mice treated with vehicle or GSK-LSD1 inhibitor. (**B**) Graphical representation of changes in tumor size over the course of the experiment. (**C**) Graphical representation of the effects LSD1 inhibition on the mean tumor volume as measured at the end of the experiment. (**D**) The FACS analysis of dissociated tumor cells isolated pre-treatment or post-treatment with GSK-LSD1 or vehicle. Single cells were stained with antibodies against the CD44 and CD24 cell surface proteins. (**E**) Quantification of FACS analysis presented in (**D**). Error bars represent SEM. *** *p* < 0.001, **** *p* < 0.0001.

As shown in Figure 5A,B, inhibitor-treated tumors grew significantly smaller than the vehicle-treated ones. In the mice that were administered GSK-LSD1, the mean tumor volume was 4-fold smaller compared to the control group (Figure 5C). More importantly, FACS analysis for the CD44-CD24 markers (Figure 5D) revealed that administration of the LSD1 inhibitor resulted in a significant reduction in the number of tumor CSCs (CD44^+^CD24^-/low^), compared to tumors before treatment initiation and to vehicle-treated ones (Figure 5E). These results represent convincing evidence that, by inhibiting LSD1, the breast CSC subpopulation was depleted, leading to tumor shrinkage in vivo.

Overall, the above data indicate that pharmacologic inhibition of LSD1 has a strong impact on stemness properties and it can lead to a significant reduction of the tumor CSC compartment both in vitro and in vivo.

### 2.7. Combination Therapy with LSD1 Inhibitors Is More Efficient in Eliminating All Tumor Cells

The above experiments suggested that LSD1 increased breast cancer cell resistance to chemotherapy, possibly through regulating the self-renewal and tumor-initiation capacities of CSCs. We also showed that LSD1 targeting led to a reduction of the tumor CSC subpopulation and significant regression in tumor size in mouse xenografts. However, breast cancer cells are known to display remarkable plasticity and spontaneously switch to a stem-like state [56]. Thus, it has been proposed that for therapies to be clinically effective and lead to complete tumor eradication, they need to combine a chemotherapeutic drug against non-CSCs and a CSC-targeting agent [14].

**Figure 6:**
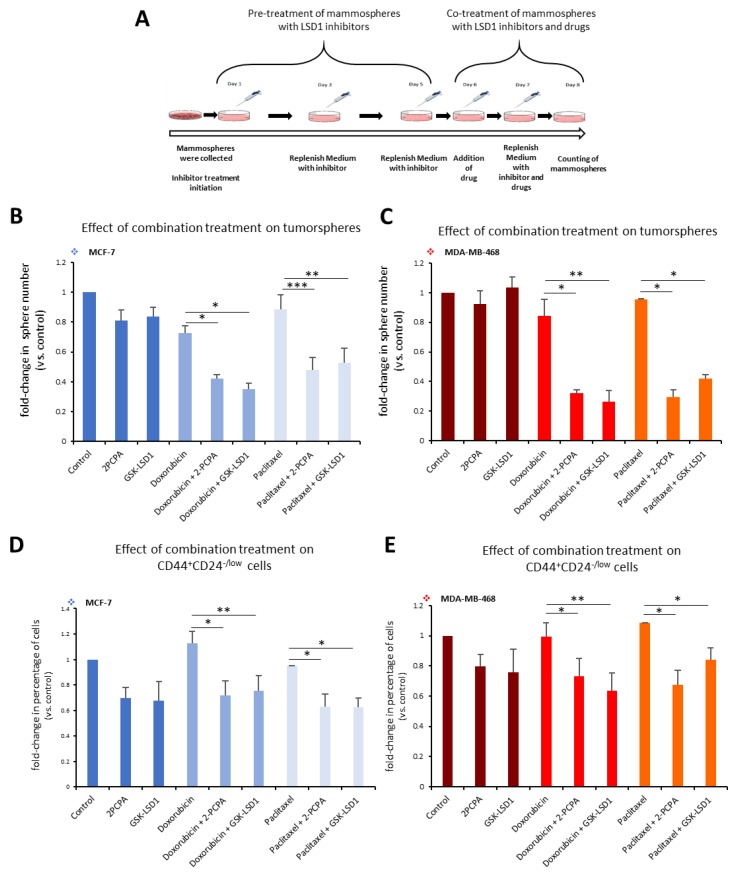
Combination treatment of breast tumorspheres with LSD1 inhibitors targets all cancer cells. (**A**) Schematic representation of combination treatment of tumorspheres with LSD1 inhibitors and an anti-cancer drug. MCF-7- and MDA-MB-468-derived tumorspheres were treated with 2-PCPA (50 μΜ) or GSK-LSD1 (2 μΜ) for 5 days. On the sixth day, doxorubicin (2.5 μΜ) or paclitaxel (15 μΜ) were added for two more days. On the last day of treatment, the number of tumorspheres was counted. (**B**) Graphical representation of the effects of combination treatment on MCF-7 tumorspheres. The dark blue panel depicts vehicle-treated, 2-PCPA- or GSK-LSD1-treated tumorspheres. The light blue panel depicts doxorubicin-treated tumorspheres, alone or in combination with an LSD1 inhibitor. The grey blue panel depicts paclitaxel-treated tumorspheres, alone or in combination with an LSD1 inhibitor. (**C**) Graphical representation of the effects of combination treatment on MDA-MB-468 tumorspheres. The dark red panel depicts vehicle-treated, 2-PCPA- or GSK-LSD1-treated tumorspheres. The red panel depicts doxorubicin-treated tumorspheres, alone or in combination with an LSD1 inhibitor. The orange panel depicts paclitaxel-treated tumorspheres, alone or in combination with an LSD1 inhibitor. (**D**) Graphical representation of FACS analysis data for the CD44^+^CD24^-/low^ CSC subpopulation in MCF-7 tumorspheres. Colors depict the same conditions described in (**B**). (**E**) Graphical representation of FACS analysis data for the CD44^+^CD24^-/low^ CSC subpopulation in MDA-MB-468 tumorspheres. Colors depict the same conditions described in (**C**). Error bars represent SEM. * *p* < 0.05, ** *p* < 0.01, *** *p* < 0.001.

Along these lines, we employed a 3D tumorsphere in vitro system composed of heterogeneous cancer cell populations, to develop a pharmacological protocol that combined the administration of an LSD1 inhibitor with a drug, so as to efficiently target all cancer cells. We used the two specific LSD1 inhibitors, 2-PCPA and GSK-LSD1, in combination with two common anti-cancer drugs, doxorubicin and paclitaxel, and applied different schemes regarding dosage, treatment time, and serial drug administration (data not shown). The final protocol we established is shown in Figure 6A. Tumorspheres generated from MCF-7 and MDA-MB-468 cell lines were pre-treated for 5 days with 2-PCPA (50 μΜ) or GSK-LSD1 (2 μΜ). On the fifth day, the chemotherapeutic drug, doxorubicin (2.5 μM) or paclitaxel (15 μM)**,** was added for 2 more days. On the last day of treatment, the number of tumorspheres was counted (Figure 6A). In MCF-7 tumorspheres, monotreatment with an LSD1 inhibitor or drug had minor impact on the number of spheres compared to vehicle-treated spheres (Figure 6B). Administration of doxorubicin alone led to a 28% decrease; pre-treatment with 2-PCPA or GSK-LSD1 followed by addition of doxorubicin led to a 58% or 65% reduction respectively, compared to the control (Figure 6B, light blue panel). Combination of paclitaxel with 2-PCPA or GSK-LSD1 led to a reduction of 52% or 47%, respectively, while paclitaxel alone resulted in a 11% decrease in the number of spheres, compared to the control (Figure 6B, grey blue panel). In all cases, the combinatory administration yielded significantly better results than the drug by itself. The effects were even more severe in the MDA-MB-468 tumorspheres (Figure 6C). When doxorubicin was combined with an LSD1 inhibitor, there was a reduction by 68% (with 2-PCPA) or 72% (with GSK-LSD1), compared to vehicle-treated spheres (Figure 6C, red panel).

Again, these numbers were significantly smaller than monotreatment with the drug, which only caused a 15% decrease in the number of spheres. Similar data were obtained with paclitaxel (Figure 6C, orange panel). The drug by itself only led to a 5% reduction in the number of MDA-MB-468 spheres, while there was a −65% decrease in their number, when they were treated both with the drug and an LSD1 inhibitor (Figure 6C, orange panel).

To confirm the synergistic action between the LSD1 inhibitors and the anti-cancer drugs, we adopted an effect-based approach and plotted our data accordingly (Figure S6). In MCF-7 tumorspheres, monotreatment alone had a minor effect on their number; however, combined treatment exceeded the additive effect (represented by the dashed lines) suggesting a synergistic action among the two pharmacological agents (Figure S6A). This was also confirmed by further experiments, where we tested different dose ratios in MCF-7 tumorspheres (Figure S6B,C). In all ratios tested, doxorubicin (Figure S6B) and paclitaxel (Figure S6C) had a synergistic effect with both LSD1 inhibitors. Similar data were obtained with the MDA-MB-468 tumorspheres (Figure S6D–F). Combined treatment using the treatment scheme shown in Figure 6 had a synergistic effect on the number of tumorspheres (Figure S6D) reducing them significantly. Different dose ratios of doxorubicin (Figure S6E) or paclitaxel (Figure S6F) with an LSD1 inhibitor acted synergistically on the tumorspheres greatly decreasing their number.

These data indicate that combinatory administration of a chemotherapeutic drug with an LSD1 inhibitor is more successful in eliminating breast tumorspheres, probably because all cancer cell populations are targeted effectively.

This was further confirmed by FACS analysis of the tumorspheres at the end of the experiment, where we monitored the CD44^+^CD24^-/low^ CSC subpopulation (Figure 6D,E). In accordance with our previous findings, neither doxorubicin nor paclitaxel alone were capable of targeting the pool of CSCs in MCF-7 tumorspheres (Figure 6D). However, addition of an LSD1 inhibitor was efficient in reducing their number by −28% in combination with doxorubicin (Figure 6D, light blue panel), or by 38% in combination with paclitaxel (Figure 6D, grey blue panel) compared to the control. Similar results were obtained with the MDA-MB-468 tumorspheres (Figure 6E). Combination of doxorubicin with 2-PCPA or GSK-LSD1 led to a reduction by 27% and 37%, respectively (Figure 6E, red panel). Addition of paclitaxel following 2-PCPA or GSK-LSD1 resulted in a decrease of 33% and 15% in the number of CSCs, respectively (Figure 6E, orange panel).

Taken together, the above data suggest that inclusion of an LSD1 inhibitor in combination treatments may be an advantageous therapeutic approach in breast cancer.

## 3. Discussion

Breast tumor heterogeneity, driven by genetic and/or epigenetic mechanisms, represents a major challenge in modern oncology, with clinicians having documented that most current therapies do not efficiently target all cancer cells [17,57]. The cells that survive treatment contribute to tumor relapse and metastasis, making it critical to comprehend the molecular basis of resistance, so that more effective therapeutic approaches against all tumor cells can be developed.

It is increasingly recognized that epigenetic mechanisms are contributing factors in the emergence of drug resistance [10], either through enhancing the plasticity of cancer cells that may adopt a chemo-resistant chromatin state [58], or by maintaining pools of cancer stem-like cells that are characterized by self-renewal and reduced sensitivity to conventional therapeutic schemes [14].

In this study, we investigated the role of the histone demethylase LSD1 in breast cancer and we provide substantial evidence that it is implicated in the development of chemoresistance, more likely, through the regulation of the cancer stem cell tumor compartment.

Following our initial findings that pretreatment of breast cancer cells with an LSD1 inhibitor rendered them more vulnerable to doxorubicin treatment, we employed knock-down and overexpression experiments and confirmed LSD1’s involvement in the chemoresistance of these cells.

In both breast cancer cell lines used, MCF-7 and MDA-MB-468, a decrease in LSD1 levels was accompanied by a reduction in the number of cells that survived after doxorubicin administration, while overexpression of the enzyme had the opposite results. To our knowledge, this is the first evidence that LSD1 is directly implicated in regulating the chemoresistance of breast cancer cells. Our results are supported by a recent study that showed that a phosphorylated form of LSD1 (p-LSD1) at Ser111 was enriched in docetaxel-resistant breast cancer cell lines and both LSD1 and p-LSD1 were upregulated in tumor cells surviving taxane-therapy in mouse xenografts [32].

Drug resistance is ascribed as a main property of CSCs, along with self-renewal and tumor-initiation. The emerging theme of LSD1 being a critical regulator of cancer stemness, corroborated both by basic research [27,28,29,30,31,32] and clinical studies associating its expression with aggressive and poorly differentiated tumors [25], prompted us to investigate more thoroughly its role in breast cancer stem cells.

The breast cancer cell lines we used to conduct our experiments—namely, MCF-7 and MDA-MB-468—contained a functional CD44^+^CD24^-/low^ cancer stem cell subpopulation, as per the published literature [59] and our own preliminary experiments had indicated (data not shown). We confirmed that CSCs contained in these cell lines had increased resistance to chemotherapy and were enriched following drug administration. The post-treatment enhancement of the CSC subpopulation is considered to be one of the underlying causes of therapy failure, with the resistant cells fueling tumor re-growth and patient relapse. Thus, our in vitro system captured the challenges presented in the in vivo tumor setting and we used it in subsequent experiments to address the role of LSD1 in bCSCs.

Our extensive transient and stable knock-down experiments produced consistent data that LSD1 depletion had a strong negative effect on the stemness potential of bCSCs in vitro. Our results are in accordance with an older study that showed that the USP28–LSD1 axis controlled CSC-like properties in human breast cancer [30]. Knock-down experiments of either enzyme led to reduced tumorsphere formation and a decrease in the CSC population in MCF-7 and BT-549 cells [30]. On our end, we provided further evidence by also overexpressing LSD1 and succeeding to enhance the self-renewal ability of bCSCs, as manifested by an increased capacity in forming tumorspheres and a boost in their numbers in vitro.

A principal property of CSCs is their increased tumor-initiation capacity. The LDA is employed as a tool to estimate CSC abundance and evaluate their self-renewal and tumorigenic potential in vivo. Our orthotopic xenotransplantation assays with increasingly diluted singe-cell preparations of parental and stably LSD1 knock-down cells indicated a devastating effect of LSD1 depletion on tumor-initiating cells. Parental MDA-MB-468 cells were able to form tumors down to the 10^5^ cell dilution; however, the LSD1-deficient cells did not yield any tumors, at any dilution, during the time period of our experiments. These results are surprising, as we would expect that some tumors would be formed, at least, with the highest inoculation numbers of the MDA-MB-468-shLSD1a cells, but this is not unprecedented. Similar data were produced from xenotransplantation assays with glioblastoma stem cell lines, where LSD1 knock-down resulted in abrogation of brain tumor formation and/or very long delays in their appearance [60,61]. In breast cancer, a previous study, using the triple-negative (TN) MDA-MB-231 cell line showed that its stable LSD1 knock-down derivative induced smaller tumors in mice compared to parental cells [62]. We used the less aggressive TN MDA-MB-468 cell line, since it was also used in our in vitro assays. The total absence of tumor formation could be due to the high knock-down efficiency in our MDA-MB-468-shLSD1a cells or it can be attributed to cell-type-specific differences among the two TN cell lines. It is plausible that the highly aggressive MDA-MB-231 cells have additional mechanisms that help them overcome the deficit in LSD1 protein.

Enzymes are leading targets for the design and development of therapeutic small molecules and numerous LSD1 inhibitors have, already, been described in the literature [63] with some of them undergoing clinical trials for different types of leukemia and solid tumors (not including breast cancer). Our experiments showed that employment of two different LSD1 inhibitors, 2-PCPA and GSK-LSD1, was successful in reducing the number of CSCs and their stemness potential in vitro, producing similar phenotypes with the LSD1 knock-down. These results substantiate the use of pharmacological agents for the specific targeting of the enzyme and the breast CSC-programs it regulates. More importantly, these results were verified in an in vitro tumorsphere system generated from breast cancer patient tumor samples, where LSD1 inhibition dramatically reduced the stemness potential of tumor CSCs. To our knowledge, these are the first data showing the effects of LSD1 inhibition on patient mammospheres and they underline the clinical significance of our findings. Finally, these data were also confirmed by in vivo experiments in mice bearing breast cancer xenografts. Administration of an LSD1 inhibitor hampered tumor growth, but the most significant finding was that it also led to a pronounced reduction of the stem-like CD44^+^CD24^-/low^ subpopulation. Overall, these experiments provide unequivocal evidence that targeting of this enzyme has a direct effect on the CSC tumor subpopulation in vivo and this may be contributing to deceleration of tumor growth.

Our data, thus far, has indicated that targeting both CSCs and non-CSCs in breast cancer should yield better therapeutic results. With this in mind, we employed a 3D in vitro tumorsphere system, as a versatile and effective tool [64], for the development and application of a combination protocol, including an LSD1 inhibitor and a chemotherapeutic drug. The main advantage of this system was that it allowed us to experiment with different administration schemes and monitor the effects on the entire tumor cell population, as reflected in the number of tumorspheres, and on the CSC subpopulation (CD44^+^CD24^-/low^), as analyzed by FACS, at the same time. It is noteworthy that both drugs tested, doxorubicin and paclitaxel, did not have a significant effect on the number of spheres, when administered as monotherapy, and neither did the LSD1 inhibitors. However, their combination was detrimental to the tumorspheres, reducing their numbers significantly. Our experiments showed a synergistic action between the anti-cancer drugs and the LSD1 inhibitors at the dose-ratios tested. Another striking finding was that the drugs alone had no effect or caused a small enrichment of the CSC subpopulation, in agreement with our previous findings. Administration of an LSD1 inhibitor was necessary for the targeting of these cells. In the protocol used, the parallel administration of LSD1 inhibitors and drugs did not have a synergistic effect on the number of CSCs. It seems that the inhibitor by itself was sufficient to produce the desired effect. However, as stated above, monotreatment with the inhibitors was not successful against the entire tumorsphere, indicating their inefficiency against more differentiated cancer cells. Therefore, both classes of agents are needed to achieve complete tumor (sphere) elimination. During the course of these experiments, a study was published that was in agreement with our data [32]. The authors showed that combination therapy, using a different LSD1 inhibitor, phenelzine, and paclitaxel, blocked chemotherapy-induced EMT and suppressed tumor growth in MDA-MB-231 mouse xenografts [32].

Collectively, our data suggest that LSD1 is a mediator of drug resistance in breast cancer cells and establish it as a key regulator of cancer stemness, since it affects the self-renewal and tumor-initiation capacity of bCSCs in vitro and in vivo. Our work provides proof-of-concept that novel therapeutic regimens, combining a conventional cytotoxic drug with a CSC-targeting agent, are likely to overcome the failure of current anti-cancer approaches to cope with breast intratumoral heterogeneity. Under this scope, LSD1 inhibitors hold promise for the future as such agents. Future studies should aim to design more specific inhibitors for the enzyme and optimize pharmacological protocols for the administration of the combined drugs.

## 4. Materials and Methods

### 4.1. Cell Lines and Pharmacological Agents

Human breast cancer cell lines, MCF-7 and MDA-MB-468, were purchased from ATCC (LCG standards) and were cultured in Dulbecco’s modified Eagle’s medium (DMEM, high glucose) supplemented with 10% fetal bovine serum and 1% penicillin/streptomycin in a humidified atmosphere of 5% CO_2_ at 37 °C. Cells were routinely passaged every 2 or 3 days and tested for mycoplasma. The LSD1 inhibitors used were 2-PCPA (trans-2-phenylcyclopropylamine) (Cayman Chemicals, cat. no. 1986) and GSK-LSD1 (Cayman Chemicals, cat. no. 16439). The drugs used were doxorubicin (Adriblastina Hydrochloride 10 mg/5 mL VIAL Pfizer) and paclitaxel (PATAXEL VIAL 30MG X5ML Vianex).

### 4.2. Transfections and Generation of Transient and Stable Knock-Down Cell Lines

A small interfering RNA (siRNA) that was published before [43] was used for transient LSD1 knock-down and was transfected into cells using Lipofectamine™ RNAiMAX Transfection Reagent (ThermoFisher Scientific) according to manufacturer’s instructions. A scrambled siRNA was used as a negative control [43]. For the generation of cell lines with stable LSD1 knock-down, two different shRNAS (sequenced provided in Supplementary Figure S4B) were cloned into pLKO.1-puro plasmid vectors, lentiviral particles were generated according to standard protocols and were used for the transduction of MCF-7 or MDA-MB-468 cell lines. After puromycin selection, the cells that survived were collected and evaluation of the LSD1 knock-down was performed by RT-PCR and Western blot analysis. For LSD1 overexpression experiments, LSD1 expression vectors were obtained from Battaglioli’s Laboratory at the Department of Biology and Genetics for Medical Sciences, University of Milan, Italy [65]. Plasmids were transfected using TransfeX™ Reagent (ATCC) for MCF-7 cells and Lipofectamine 3000 Reagent (Thermo Fisher Scientific) or X-tremeGENE™ HP DNA Transfection Reagent (Roche) for MDA-MB-468 cells according to manufacturer’s instructions. An empty vector was used as control. Evaluation of the LSD1 knock-down was performed by RT-PCR and Western blot analysis. Densitometric analysis of all Western blots was shown in Figure S7.

### 4.3. Mammosphere Formation Assay

Cells at an early passage were resuspended in mammosphere medium (DMEM/F12 supplemented with B27 (Gibco), EGF (20 ng/mL, Immunotools), and FGF (20 ng/mL Immunotools)) and were seeded into 6 well plates, coated with poly-2-hydroxyethyl methacrylate (pHema, SIGMA-Life Science, St. Louis, MO, USA) dissolved in 95% ethanol (EtOH, 20 mg/mL). Mammospheres were grown between 7–10 days depending on the cell line. Only spheres with a diameter over 50 μm were counted. The mammopshere formation efficiency (MFE) was calculated based on the following formula: (number of mammospheres per well/number of cells seeded per well) × 100 [48].

### 4.4. Flow Cytometry

Breast cancer cells were harvested (or dissociated from mammospheres) and were incubated with anti-CD44-PE (Cat. No. 550989) and anti-CD24-FITC (Cat. No. 560992) conjugated antibodies (both from BD Biosciences, San Jose, CA, USA) for 20 minutes at 4 °C in the dark. The staining of the cells was followed by two washes with PBS-2% FBS. Finally, the cells were centrifuged (1500 rpm, 5 min, 4 °C) and then resuspended in 200 μL PBS-2% FBS. Analyses were then performed using the BD FACS Aria II-BD Biosciences. Gates for fluorescence fractionations were established using unstained and isotype controls, PE-IgG (Cat. No: 21275514) and FITC-IgG (Cat. No. 21815013) both from Immunotools.

Cells from xenografts were also stained with the PerCP/Cy5.5 anti-mouse H-2Kd (Biolegend, Cat. No: 116628) for the exclusion of mouse cells from the xenograft analysis. For these cells, FACS analysis was performed on the Cytomics FC500 (BECKMAN COULTER).

### 4.5. Cell Proliferation Assays

For the cell proliferation assays and IC_50_ calculation, the Incucyte Zoom system (Essen BioScience, Hertfordshire, United Kingdom) and software was used. The MCF-7 and MDA-MB-468 cells were seeded in triplicate into 96 well plates at low confluency and pre-treated with GSK-LSD1 (0.5 μΜ) for five days. On the sixth day, serial dilutions of doxorubicin (0.01–5 μΜ) were added for 48 h. The Incucyte Zoom live-cell imaging system was used to obtain phase contrast images of the cells every 12 h for a total of 48 h and confluency was determined using the associated software. The relative increase in cell number values was generated for every well using the confluence readings relative to the starting confluence. The Graphpad Prism 8.01 software was used to calculate the IC_50_ under the different conditions.

### 4.6. Pharmacological Treatment

To examine the chemoresistance of CSCs contained in the MCF-7 and MDA-MB-468 cell lines, breast cancer cells, at early passages, were seeded in duplicates in multi-well cell culture plates, so that the cells would reach a confluency of 40–60% the first day of treatment. The MCF-7 cells were treated with 1–10 μΜ doxorubicin for 48 h or 1–25 μΜ paclitaxel for 6 days. The MDA-MB-468 cells were treated with 2.5 or 5 μΜ doxorubicin for 48 h or 10–25 μΜ paclitaxel for 6 days. On the last day of treatment, the number of live cells was recorded.

Treatment with the LSD1 inhibitors for the calculation of the MFE was performed with increasing doses of 2-PCPA (5–50 μΜ) and GSK-LSD1 (0.1–2 μΜ), based on previously published literature [30,42]. The MFE was calculated at different time points (3–10 days) and FACS analysis was also performed to monitor the CD44^+^CD24^-/low^ CSC subpopulation. A dose-response effect was observed (data not shown). The highest MFE and the most pronounced reduction in CSCs was achieved with 50 μΜ 2-PCPA and 2 μΜ GSK-LSD1 at 7 days and these concentrations were used in subsequent experiments.

For the combination treatment of tumorspheres with LSD1 inhibitors and drugs, 1st generation tumorspheres were generated, as described above, and pre-treated with 2-PCPA (50 μΜ) or GSK-LSD1 (2 μΜ) for 5 days, before the addition of doxorubicin (2.5 μΜ) or paclitaxel (15 μΜ) for 2 more days. The concentrations used were selected so as to minimize the inhibitor/drug dose and maximize responses based on our preliminary experiments (data not shown).

### 4.7. Immunoblot Analysis

For Western blot analysis, total protein quantitation was performed using the BCA Protein Assay (ThermoScientific, Waltham, MA, USA). The antibodies used were anti-KDM1/LSD1 antibody (Abcam ab17721-1:1000), anti-TUBULIN (Developmental Studies Hybridoma Bank (DSHB)-E7)-1:2000), anti-Actin (a.a. 50-70, clone C4 Millipore U.S.A. MAB1501- 1:10000), anti-rabbit IgG-HRP-linked (Cell Signaling 7074-1:2000), and anti-mouse IgG-HRP-linked (Cell Signaling 7076-1:2000).

### 4.8. Immunohistochemistry

Representative formalin-fixed and paraffin-embedded (FFPE) tissue sections from ten patients with triple-negative breast invasive carcinomas (TNBC) of no special type (NST) (all female, median age: 62) were processed for immunohistochemical study. The tissue blocks were collected from the archives of the Department of the Pathology of the University of Ioannina. The vast majority of them (9 out of 10) were grade 3 and one was grade 2. Double staining for CD44 and LSD1 was performed using 4 μm thick full sections in a semi-automated staining system (Autostainer link 48 DAKO). Primaries antibodies were CD44 (dilution 1:1000, Invitrogen, CD44 MA5-13890) and LSD1 (dilution 1:80, Abcam ab17721-1:1000). The CD44 showed membranous immunostaining (chromogenic substrate: Magenta, DAKO), whereas the LSD1 nuclear (chromogenic substrate: DAB, DAKO). Each case was evaluated for the percentage of the LSD1- and CD44-positive cells, as well as the intensity of the LSD1 immunostaining. The nuclear immunostaining was semi-quantitatively evaluated as follows: negative (0), weak (1), moderate (2), and strong (3).

### 4.9. Patient Tumor Dissociation and Tumorsphere Culture

Human breast cancer tissue was obtained from breast cancer patients undergoing mastectomy for therapeutic purposes after their informed consent. The study was approved by the Scientific Board of the General University Hospital of Ioannina (5/260214, Ioannina, Greece). Tumor samples were excised by an anatomical pathologist and were transferred to the lab, for further processing, in M199 medium, supplemented with antibiotics (penicillin/streptomycin, 1%), on ice and within an hour after surgery. After rinsing with PBS, tumor samples were minced and then enzymatically digested with Collagenase type III (220 U/mL) for 2 h at 37 °C, under rotation. Subsequently, samples were filtered first through a 100 μm and then a 40 μm cell strainer and were spinned down. The cell pellet was finally resuspended in mammosphere medium and plated in polyHema-coated plates, as described above, where tumorspheres were grown for 7 to 10 days.

### 4.10. In Vivo Experiments with Breast Cancer Xenografts in Mice

Female non-obese diabetic/severe combined immunodeficiency (NOD/SCID) and NSG (NOD/SCID *γ*-irradiated) mice were housed at the Animal House Facility of the Foundation for Biomedical Research Foundation of the Academy of Athens (Athens, Greece) under veterinarian supervision and in full compliance with FELASA (Federation of Laboratory Animal Science Associations) recommendations. The animals were bred in individually ventilated cages under specific pathogen-free conditions. All procedures for care and treatment of animals were approved by the Institutional Committee on Ethics of Animal Experiments (protocol number 1763 04/04/17) and the Greek Ministry of Agriculture.

Female NOD/SCID mice were orthotopically transplanted with MDA-MB-468 cells. The 1 × 10^7^ cells per 100 μL of medium or PBS were directly injected in the fat pad of 5 week old mice according to published protocols [66,67,68]. Upon palpable tumor formation, tumor size was measured twice a week with a caliper. When tumors reached ~0.4 mm, the mice were divided randomly in two cohorts. In one cohort (*n* = 6), oral GSK–LSD1 administration was performed (gavage) with 1 μg/kg dose for 5 days per week (3 consecutive days then a one-day break, two consecutive days and a one-day break). The other cohort (*n* = 5) received vehicle control in the same fashion. The treatment lasted for 3 weeks. At the end of the experiment, all the mice were euthanized, and the excised tumors were measured, weighted, and processed for FACS analysis. In particular, each tumor was minced and enzymatically dissociated with Collagenase/Hyaluronidase in DMEM/F12 medium (37 °C for 2–3 h with occasional vortexing) until complete dissociation. Then the cells were washed twice with ice cold PBS and centrifugation at 5000 rpm. The cell pellet was dissolved in 500 μL of Dispase II (10 mg/mL) and DNase (0.1 mg/mL) for one minute with consecutive pipetting and then passing through an 40 μm cell strainer. After another two PBS-5%FBS washes, the cells were counted and the appropriate number was incubated with the CD44, CD24, and anti-mouse H-2kd-PerCP/Cy5.5 (BioLegend, Cat. No. 116628) antibodies.

For the in vivo LDA assessment, female NSG mice were utilized. Cohorts of 5 mice/group were labeled and orthotopically transplanted with the appropriate number of MDA-MB-468 or MDA-MB-468-shLSD1a cells (one site per mouse). Each group was transplanted with 5 × 10^6^, 10^6^, 10^5^ and 10^4^ MDA-MB-468 cells and 5 × 10^6^, 10^6^, and 10^5^ MDA-MB-468-shLSD1a cells through direct injection in one fat pad of 5 week-old littermates. All mice were monitored for palpable tumor formation every week for a total of 14 (parental cells) or 18 (knock-down cells) weeks.

### 4.11. Statistical Analysis

Data presented are of at least three independent biological experiments, unless otherwise stated, and they are expressed as mean ±SE. An unpaired two-tailed Student’s *t*-test was used for statistical analysis of the in vitro experiments. A two-sample unequal variance Student’s *t*-test was used for the in vivo xenotransplantation assays.

## 5. Conclusions

In conclusion, our data show that LSD1 is a main regulator of the key properties of bCSCs, as it plays an important role in their self-renewal and tumor-initiation capacity in vitro and in vivo. We also demonstrated that LSD1 mediates drug resistance in breast cancer cells. Our data suggest that this may be due to the fact of its role in the maintenance of a pool of functional bCSCs. We also provide strong experimental data that LSD1 is a prime candidate for the development of CSC-targeted therapies. Lysine-specific demethylase 1 inhibitors could be used in combination therapeutic schemes along with anti-cancer drugs to target all tumor subpopulations and achieve complete tumor elimination in breast cancer.

## Figures and Tables

**Table 1:** Limiting dilution assays with parental and LSD1 knock-down MDA-MB-468 breast cancer cells in mice.

Cell Line	No. of Cells Inoculated	No. of Tumors Formed	Tumor Palpation (Weeks)
ΜDΑ-ΜΒ-468	5 × 10^6^	5/5	4–5 w
	1 × 10^6^	5/5	10–11 w
	1 × 10^5^	4/5	12–14 w
	1 × 10^4^	0/5	14 w
MDA-MB-468-shLSD1a	5 × 10^6^	0/5	18 w
	1 × 10^6^	0/5	18 w
	1 × 10^5^	0/5	18 w

## Supplementary Materials

The following are available online at https://www.mdpi.com/2072-6694/11/10/1585/s1. Figure S1: Oncomine analyses of breast cancer databases; Figure S2: Western blot analysis of protein lysates after siRNA mediated knock-down of LSD1; Figure S3: Treatment with anticancer drugs enriches the bCSC sub-population; Figure S4: LSD1 regulates the stemness properties of bCSCs, Figure S5. Representative images from orthotopic xenotransplantation assays, Figure S6. Synergistic action between LSD1 inhibitors and anti-cancer drugs in 3D tumorspheres. Table S1: Immunohistochemistry results for CD44 and LSD1 in specimens from 10 TNBC patients. Figure S7. Densitometric analysis of all Western blots.
