# Supplementary material for: The Histone Demethylase LSD1/ΚDM1A Mediates Chemoresistance in Breast Cancer via Regulation of a Stem Cell Program
Source: Cancers (Basel). 2019 Oct 17;11(10):1585. doi: 10.3390/cancers11101585 (PMC6827056; doi:10.3390/cancers11101585)
Supplement: Supplementary file 1 [file cancers-11-01585-s001.pdf]

Supplementary Materials

The histone demethylase LSD1/KDM1A mediates chemoresistance in breast cancer via regulation of a stem cell program

John Verigos, Panagiotis Karakaidos, Dimitris Kordias, Alexandra Papoudou-Bai, Zoi Evangelou, Haralampos V. Harissis, Apostolos Klinakis and Angeliki Magklara

Figure S1. Supplementary Figure 1: Oncomine analyses of breast cancer databases. Box plots show that LSD1 is overexpressed in aggressive breast carcinomas: **(A)** Gluck breast cancer database [38]. Invasive breast carcinomas vs. Normal, fold-change=1.7 (p<0.005). **(B)** Curtis breast cancer database [39]. Invasive Ductal Breast Carcinoma vs. Normal, fold-change = 1.328 (p <0.005) and Invasive Lobular Breast Carcinoma vs. Normal, fold-change = 1.214 (p <0.005). **(C)** Sorlie breast cancer database [40] and **(D)** Desmedt database [41] grouped by tumor grade. There is a trend high grade tumors to exhibit higher LSD1 expression. Numbers in parentheses indicate the number of samples. The y-axis represents log2 median-centered intensity (normalized expression). Shaded boxes represent the interquartile range (25th–75th percentile). Whiskers represent the 10th–90th percentile. The bars denote the median. (adapted from www.oncomine.org)

Figure S2. Western blot analysis of protein lysates after siRNA mediated knock-down of LSD1 in **(A)** MCF-7and (**B)** MDA-MB-468 cells. Total protein lysate was isolated 5 days post-transfection. Scramble siRNA transfected cells served as mock control. Western blot analysis of protein lysates after overexpression of LSD1 in **(C)** MCF-7 and **(D)** MDA-MB-468 cells. The protein lysates were collected 3 days post-transfection. Cells transfected with empty vector served as control.

Figure S3. Treatment with anticancer drugs enriches the bCSC sub-population. (A) MCF-7 and MDA-MB-468 cells were treated with 2.5 μM Doxorubicin (2 days) or 15 μM Paclitaxel (6 days). On the last day of treatment the number of live cells was counted. **(B)** The surviving cells were subjected to FACS analysis for the CD44 and CD24 surface markers and quantitation of the data is shown. Vehicle-treated cells served as control (set to 1).

Figure S4. LSD1 regulates the stemness properties of bCSCs (A) Western blot analysis of protein lysates after siRNA mediated knock-down of LSD1 in MCF-7 and MDA-MB-468 cells. Total protein lysate was isolated 7 days post-transfection. **(B)** shRNAs sequences used for LSD1 stable knock-down. **(C)** Western blot analysis of total protein lysates for shLSD1 knock-down stable cell lines. **(D)** Effect of LSD1 stable knock-down on the M.F.E. MCF-7_shLSD1b and parental cells were cultured under mammosphere forming conditions for 7 days. **(E)** Graphic representation of FACS analysis data for mammospheres derived from MCF7_shLSD1b cells. Parental MCF-7 cells served as control. **(F)** Representative images of mammospheres derived from parental (n=7) or stable LSD1 knock-down (n=3) MCF-7 cells and parental (n=2) or stable LSD1 knock-down MDA-MB-468 cells (n=1) after 7 days in culture (enlarged images of pictures presented in Figure 2D). **(G)** Western blot analysis of protein lysates after overexpression of LSD1 in MCF-7 and MDA-MB-468 cells. Cells transfected with empty vector served as control. **(H)** Representative images of mammospheres derived from control (n=4) or LSD1-overexpressing (n=9) MCF-7 cells and control (n=3) or LSD1-overexpressing (n=6) MDA-MB-468 cells after 7 days in culture (enlarged images of pictures presented in Figure 2H). Data of at least 2 independent biological experiments are shown. Error bars represent SEM., **: $p < 0.05$.

Figure S5. Representative images from orthotopic xenotransplantation assays. Increasingly diluted singe-cell preparations of parental **(A-D)** or stably LSD1 knock-down cells **(H-J)** MDA-MB-468 cells were injected into mice. Mice injected with **(A)** $5x10^6$, **(B)** $1x10^6$ or **(C)** $1x10^5$ parental MDA-MB-468 cells formed tumors. **(D)** The highest dilution of $1x10^4$ MDA-MB-468 cells failed to yield any tumors. **(E-G)** Enlarged images of tumors shown in A-C respectively. Mice injected with **(H)** $5x10^6$, **(I)** $1x10^6$ or **(J)** $1x10^5$ MDA-MB-468-shLSD1a cells failed to form any tumors during the course of the experiment (18 weeks).

Figure S6 Synergistic action between LSD1 inhibitors and anticancer drugs in 3D tumorspheres. (A) MCF-7 derived tumorspheres were treated with 2-PCPA (50 μM) or GSK-LSD1 (2 μM) for 5 days. On the sixth day, doxorubicin (2.5 μM) or paclitaxel (15 μM) were added for 2 more days. Graphic representation of the negative effects of mono- and combination treatment on the number of MCF-7 tumorspheres (control is set to zero). The dark blue panel depicts the effects of 2-PCPA or GSK-LSD1. The light blue panel depicts the effects of doxorubicin alone or in combination with an LSD1 inhibitor. The grey blue panel depicts the effects of paclitaxel alone or in combination with an LSD1 inhibitor. The dashed lines represent the additive effects of the two agents. The combination effect is higher than the additive suggesting synergy between the two drugs. **(B-C)** MCF-7 derived tumorspheres were treated with 2-PCPA (50 μM) or GSK-LSD1 (2 μM) for 5 days, on the sixth day, different concentrations of doxorubicin (0.5-5 μM) or paclitaxel (5-25 μM) were added for 2 more days. The last day of treatment the number of tumorspheres was counted. Graphic representation of the negative effects on the number of tumorspheres for Doxorubicin **(B)** and Paclitaxel **(C)** alone or in combination with 2-PCPA or GSK-LSD1. The dashed lines represent the additive effects of the two agents. The combination effect is higher than the additive suggesting synergy between the two drugs. **(D)** Graphic representation of the negative effects of combination treatment on MDA-MB-468 tumorspheres following the protocol mentioned above. The dark red panel depicts the effects of 2-PCPA or GSK-LSD1. The red panel depicts the effects of doxorubicin alone or in combination with an LSD1 inhibitor. The orange panel depicts the effects of paclitaxel alone or in combination with an LSD1 inhibitor. The dashed lines represent the additive effects of the two agents. The combination effect is higher suggesting synergy between the two drugs. **(E-F)** Graphic representation of the negative effects on the number of tumorspheres for Doxorubicin **(E)** and Paclitaxel **(F)** alone or in combination with 2-PCPA or GSK-LSD1. The dashed lines represent the additive effect of the two agents. The combination effect is higher suggesting synergy between two drugs. Representative results from one experiment performed in two biological independent replicates with similar results are shown in Figures B, C, E, F. Error bars represent SEM.

Figure S7. Densitometric analysis of all western blots

Table S1. Immunohistochemistry results for CD44 and LSD1 in specimens from 10 (Triple Negative Breast Cancer (TNBC) patients. Double staining for CD44 and LSD1/KDM1A was performed in Representative formalin-fixed and paraffin-embedded (FFPE) tissue sections from ten patients with TNBC. Each case was evaluated for the percentage of the LSD1/KDM1A and CD44-positive cells as well as the intensity of the LSD1/KDM1A immunostaining. The nuclear immunostaining was semi-quantitatively evaluated as follows: negative (0), weak (1), moderate (2) and strong (3).

Figure S1

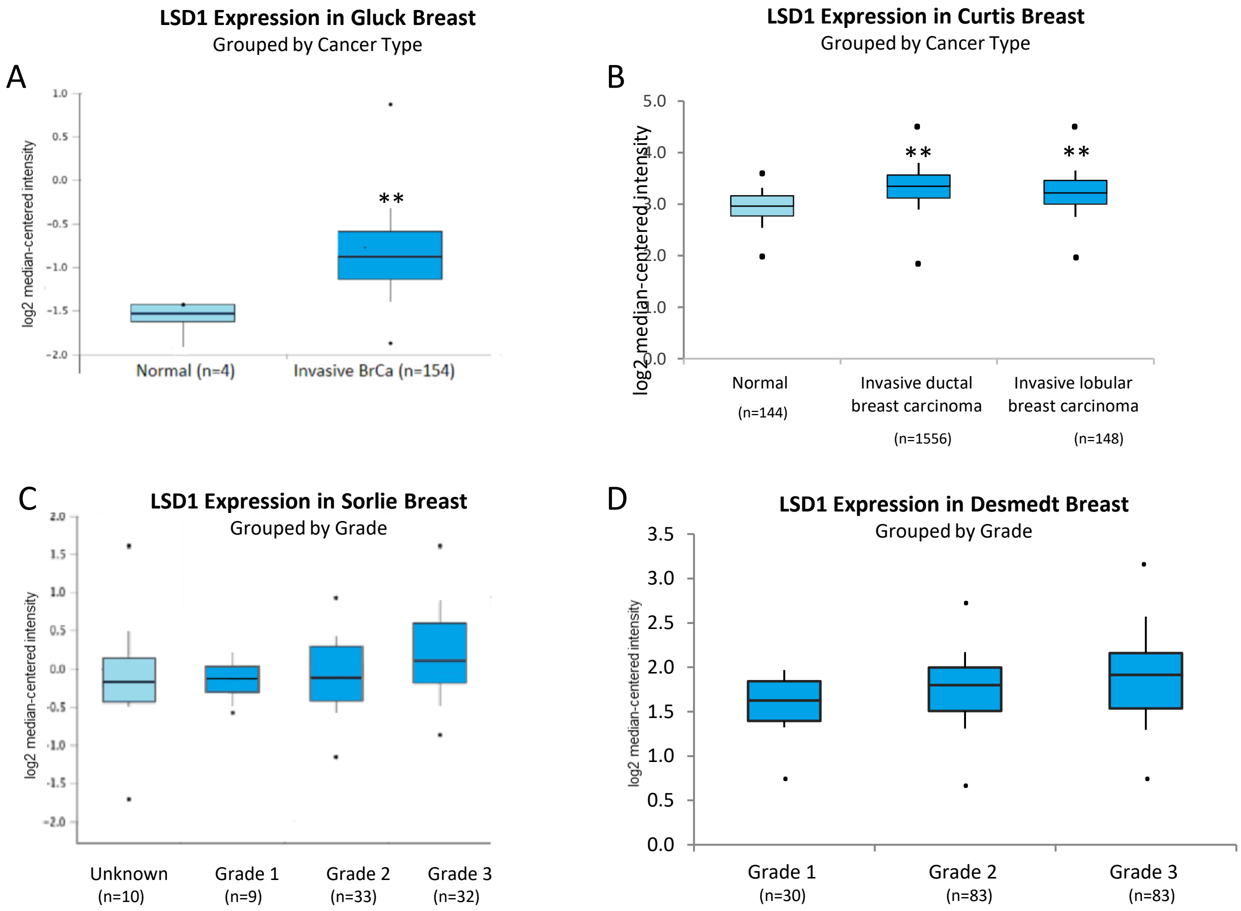
LSD1 Expression in Gluck Breast
Grouped by Cancer Type
A
log2 median-centered intensity
1.0
0.5
0.0
-0.5
-1.0
-1.5
-2.0
**
Normal (n=4)
Invasive BrCa (n=154)
LSD1 Expression in Curtis Breast
Grouped by Cancer Type
B
log2 median-centered intensity
5.0
4.0
3.0
2.0
1.0
0.0
**
**
Normal
(n=144)
Invasive ductal breast carcinoma
(n=1556)
Invasive lobular breast carcinoma
(n=148)
C
LSD1 Expression in Sorlie Breast
Grouped by Grade
log2 median-centered intensity
2.0
1.5
1.0
0.5
0.0
-0.5
-1.0
-1.5
-2.0
Unknown
(n=10)
Grade 1
(n=9)
Grade 2
(n=33)
Grade 3
(n=32)
D
LSD1 Expression in Desmedt Breast
Grouped by Grade
log2 median-centered intensity
3.5
3.0
2.5
2.0
1.5
1.0
0.5
0.0
Grade 1
(n=30)
Grade 2
(n=83)
Grade 3
(n=83)

Figure S2

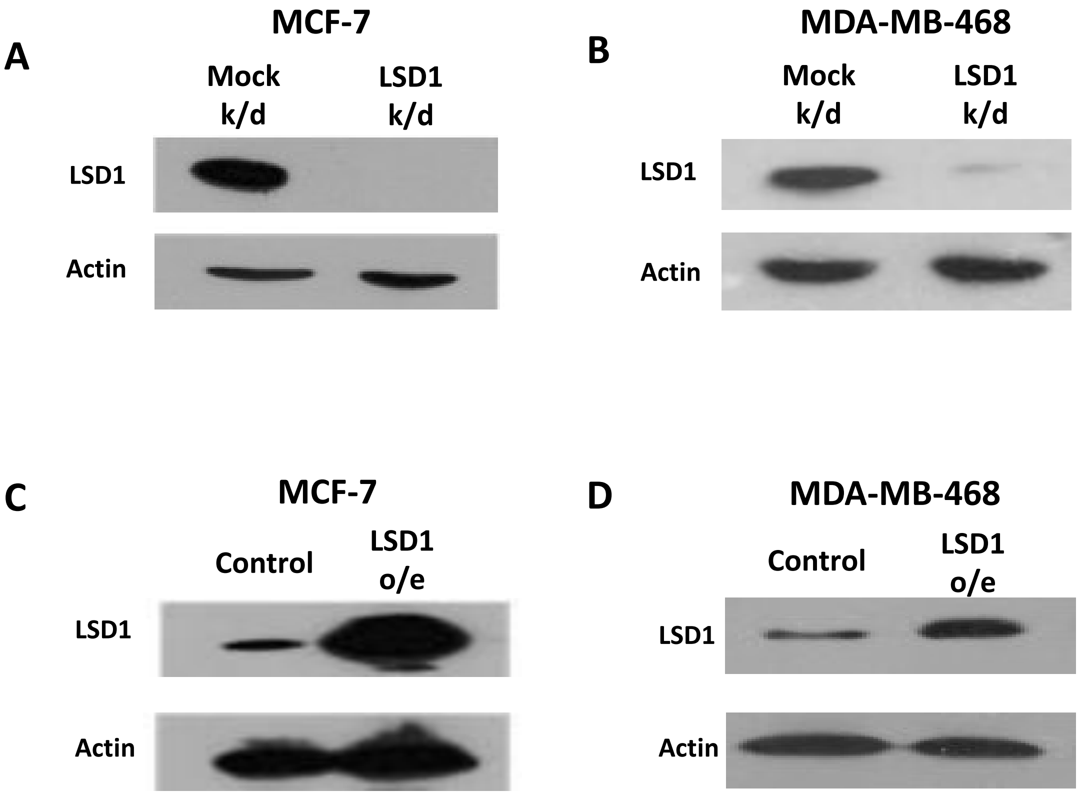
A
MCF-7
Mock k/d
LSD1 k/d
LSD1
Actin
B
MDA-MB-468
Mock k/d
LSD1 k/d
LSD1
Actin
C
MCF-7
Control
LSD1 o/e
LSD1
Actin
D
MDA-MB-468
Control
LSD1 o/e
LSD1
Actin

Figure S3

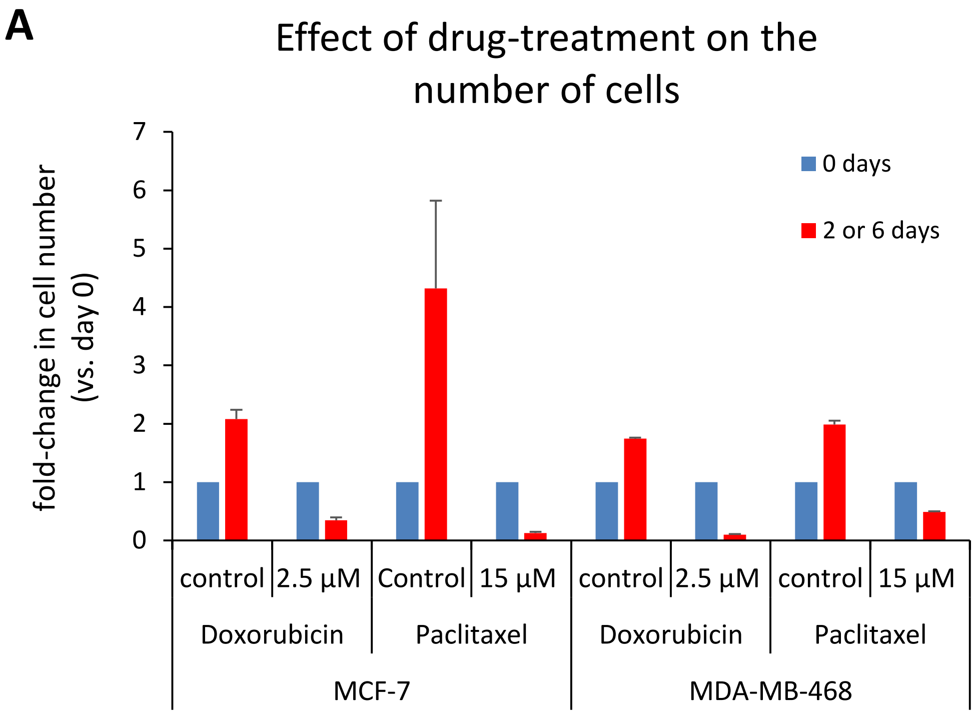
A
Effect of drug-treatment on the number of cells
fold-change in cell number (vs. day 0)
0
1
2
3
4
5
6
7
0 days
2 or 6 days
control
2.5 µM
Control
15 µM
control
2.5 µM
control
15 µM
Doxorubicin
Paclitaxel
Doxorubicin
Paclitaxel
MCF-7
MDA-MB-468

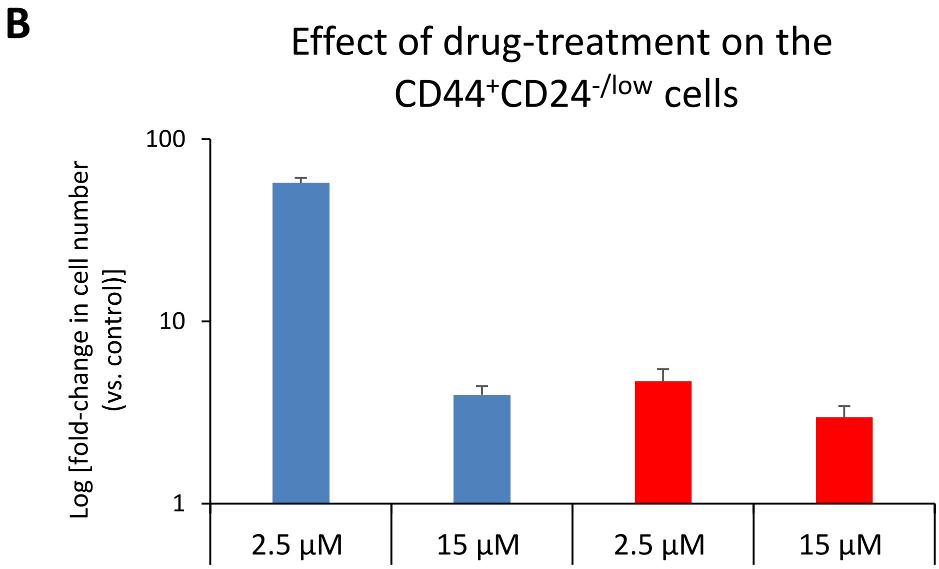
B
Effect of drug-treatment on the CD44+CD24-/low cells
Log [fold-change in cell number (vs. control)]
1
10
100
2.5 µM
15 µM
2.5 µM
15 µM

Figure S4

A

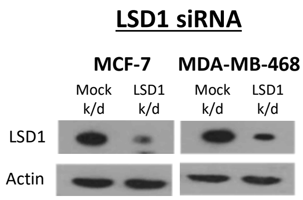

B

shRNAs		
	Sequence 5'-3'	
shLSD1a	sense 5'	CCGGGCCTAGACATTAAACTGAATACCATGGTATTCAGTTTAATGTCTAGGCTTTTTG
	Anti-sense 5'	AATTCAAAAAGCCTAGACATTAAACTGAATACCATGGTATTCAGTTTAATGTCTAGGC
shLSD1b	sense 5'	CCGGAACACAAGGAAAGCTAGAAGACCATGGTCTTCTAGCTTTCCTTGTGTTTTTTTG
	Anti-sense 5'	AATTCAAAAAAACACAAGGAAAGCTAGAAGACCATGGTCTTCTAGCTTTCCTTGTGTT

C

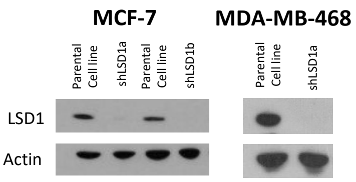

D

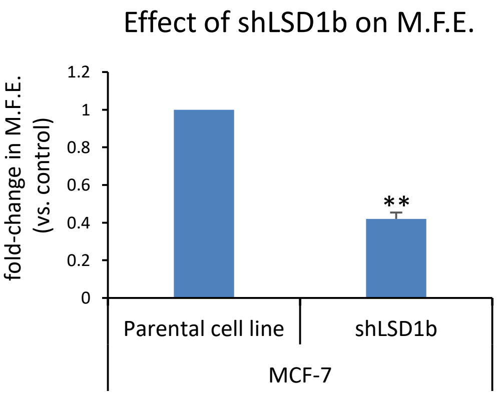

E

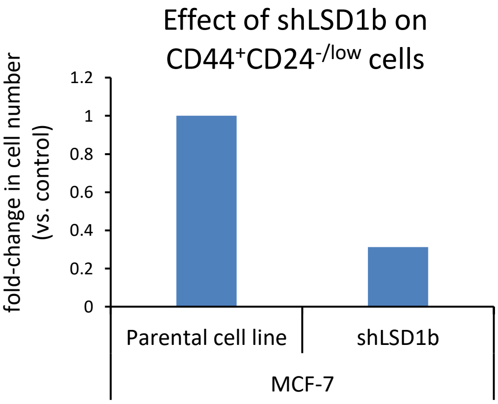

G

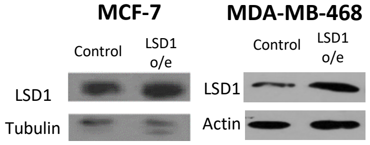

F

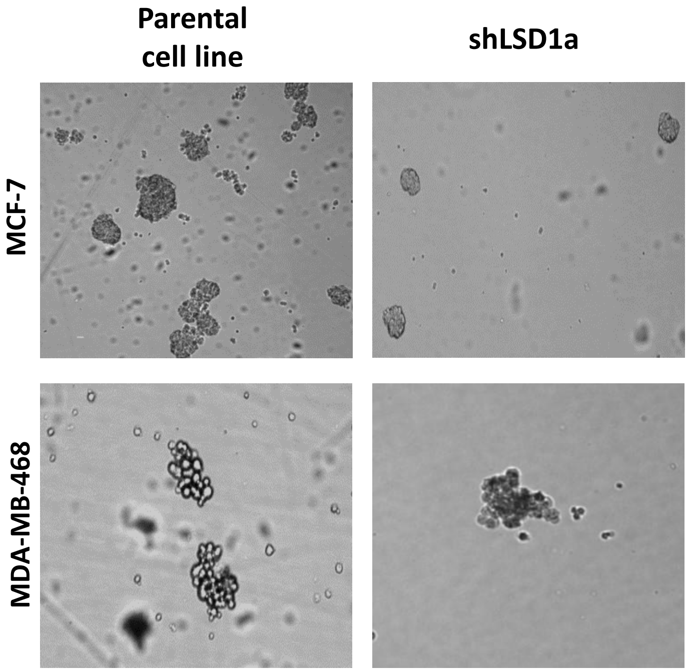

H

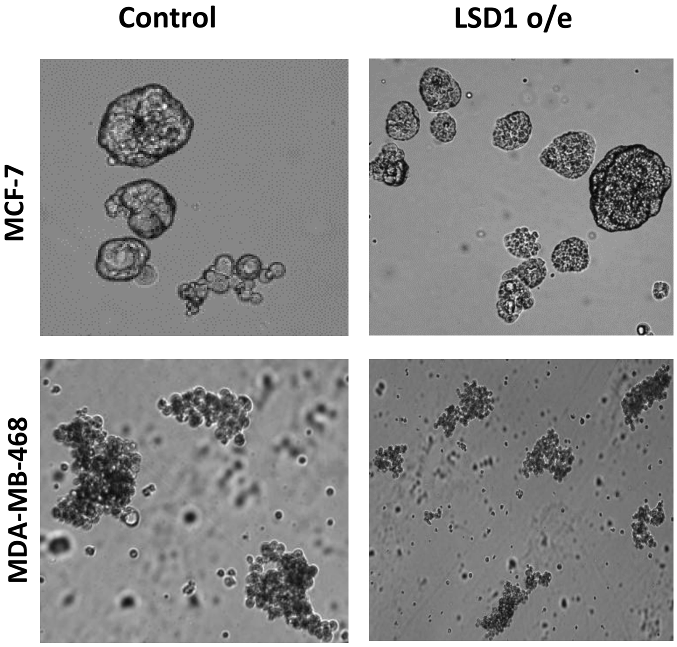

Figure S5

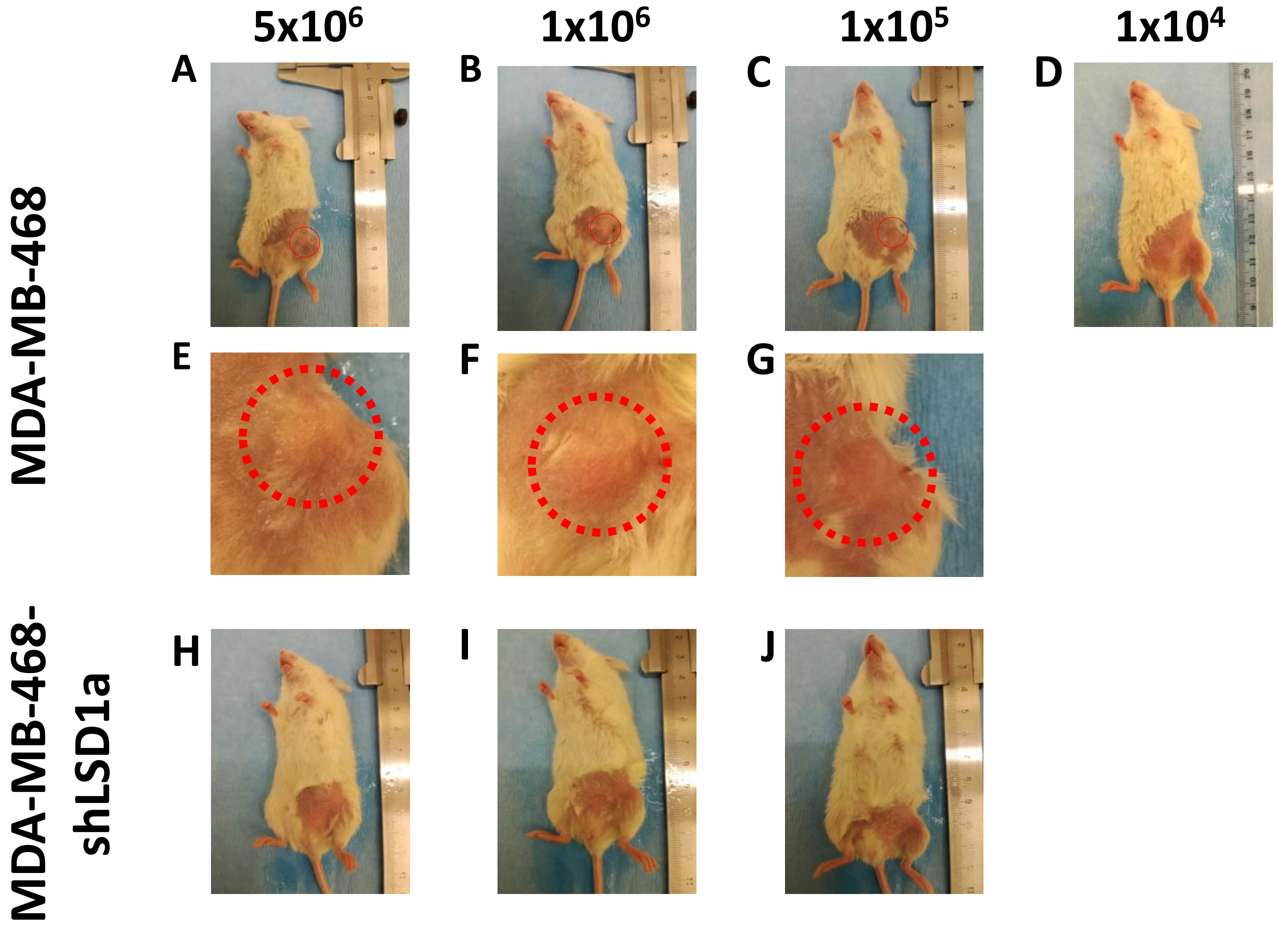
5x10^{6}
1x10^{6}
1x10^{5}
1x10^{4}
A
B
C
D
E
F
G
H
I
J
MDA-MB-468
MDA-MB-468-shLSD1a

Figure S6

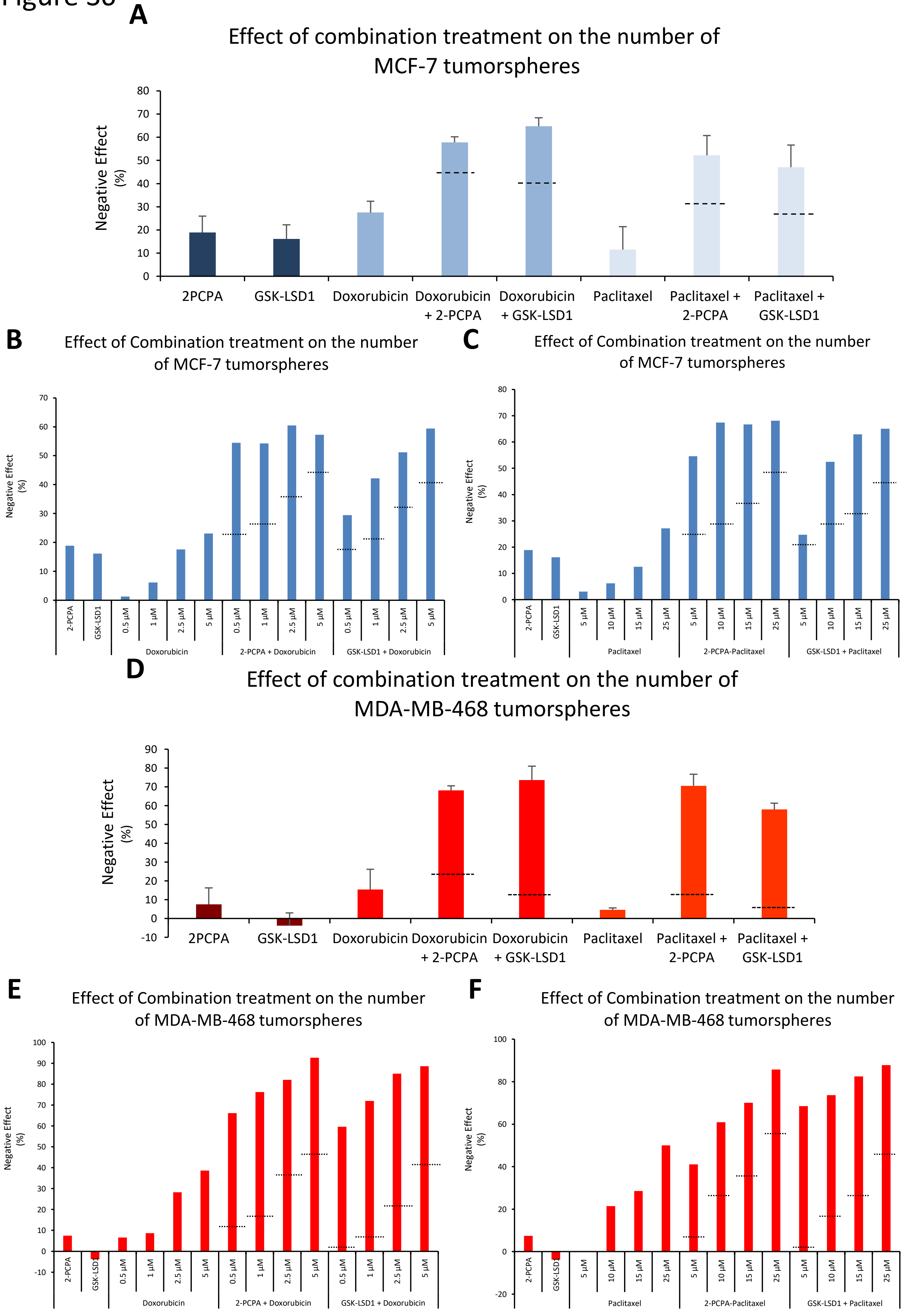
A
Effect of combination treatment on the number of MCF-7 tumorspheres
Negative Effect (%)
2PCPA
GSK-LSD1
Doxorubicin
Doxorubicin + 2-PCPA
Doxorubicin + GSK-LSD1
Paclitaxel
Paclitaxel + 2-PCPA
Paclitaxel + GSK-LSD1
B
Effect of Combination treatment on the number of MCF-7 tumorspheres
Negative Effect (%)
2-PCPA
GSK-LSD1
0.5 µM
1 µM
2.5 µM
5 µM
Doxorubicin
2-PCPA + Doxorubicin
GSK-LSD1 + Doxorubicin
C
Effect of Combination treatment on the number of MCF-7 tumorspheres
Negative Effect (%)
5 µM
10 µM
15 µM
25 µM
Paclitaxel
2-PCPA-Paclitaxel
GSK-LSD1 + Paclitaxel
D
Effect of combination treatment on the number of MDA-MB-468 tumorspheres
Negative Effect (%)
2PCPA
GSK-LSD1
Doxorubicin
Doxorubicin + 2-PCPA
Doxorubicin + GSK-LSD1
Paclitaxel
Paclitaxel + 2-PCPA
Paclitaxel + GSK-LSD1
E
Effect of Combination treatment on the number of MDA-MB-468 tumorspheres
Negative Effect (%)
Doxorubicin
2-PCPA + Doxorubicin
GSK-LSD1 + Doxorubicin
F
Effect of Combination treatment on the number of MDA-MB-468 tumorspheres
Negative Effect (%)
Paclitaxel
2-PCPA-Paclitaxel
GSK-LSD1 + Paclitaxel

Figure S7

Figure S2

LSD1 knock-down **LSD1 overexpression**

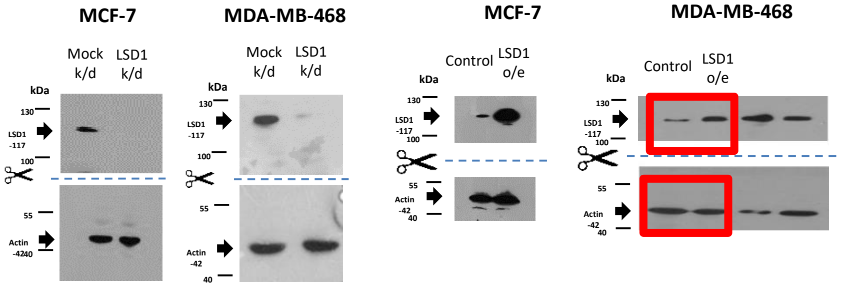

Experiment	Cell line	Sample	LSD1 (Density)	Actin (Density)
LSD1 knock-down AND Doxorubicin Treatment	MCF-7	Mock-k/d	41861.97	34743.50
		LSD1 k/d	431.85	43587.77
	MDA-MB 468	Mock-k/d	59958.02	47271.32
		LSD1 k/d	3349.45	50928.1
LSD1 OVER AND Doxorubicin Treatment	MCF-7	Control	3297.43	9513.23
		LSD1 o/e	23890.17	13790.61
	MDA-MB 468	Control	14228.522	24964.92
		LSD1 o/e	46836.472	28103.02

Figure S4

LSD1 siRNA **LSD1 overexpression**

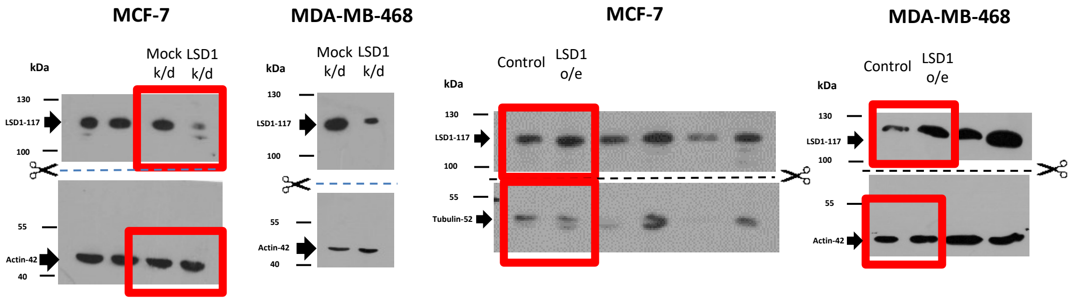

Experiment	Cell line	Sample	LSD1 (Density)	Actin (Density)
LSD1 siRNA mediated knock-down	MCF-7	Mock-k/d	23432.89	25113.63
		LSD1 k/d	5003.59	25577.34
	MDA-MB 468	Mock-k/d	23576.34	15659.39
		LSD1 k/d	5375.67	20987.02
LSD1 overexpression	MCF-7	Control	16029.85	17762.80
		LSD1 o/e	23187.41	15758.99
	MDA-MB 468	Control	8977.46	30712.05
		LSD1 o/e	27867.36	37868.85

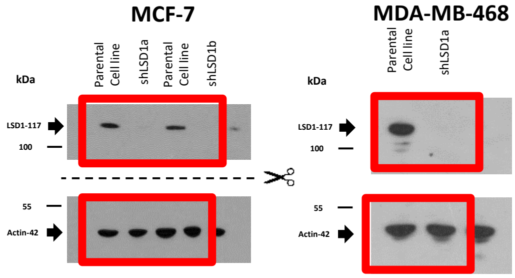

Experiment	Cell line	Sample	LSD1 (Density)	Actin (Density)
LSD1 stable knock-down	MCF-7	parental cell line	20162.27	15088.93
		shLSD1a	143.02	14772.88
		parental cell line	16337.54	20513.88
		shLSD1b	325.7	18061.76
	MDA-MB 468	parental cell line	38558.28	38757.28
		shLSD1a	-	36904.43

Table S1

CASE	Grade	CD44$^+$		LSD1/KDM1A				
				Intensity	0	1	2	3
1	2	% non-neoplastic cells	<1	% non-neoplastic cells	30	70	0	0
		% neoplastic cells	40	% neoplastic cells	5	15	30	50
2	3	% non-neoplastic cells	<1	% non-neoplastic cells	10	40	30	20
		% neoplastic cells	100	% neoplastic cells	0	10	30	60
3	3	% non-neoplastic cells	<1	% non-neoplastic cells	50	40	10	0
		% neoplastic cells	99	% neoplastic cells	1	39	40	20
4	3	% non-neoplastic cells	<1	% non-neoplastic cells	70	30	0	0
		% neoplastic cells	85	% neoplastic cells	10	60	25	5
5	3	% non-neoplastic cells	10	% non-neoplastic cells	30	40	20	10
		% neoplastic cells	100	% neoplastic cells	0	40	35	25
6	3	% non-neoplastic cells	1	% non-neoplastic cells	60	40	0	0
		% neoplastic cells	95	% neoplastic cells	5	35	40	20
7	3	% non-neoplastic cells	30	% non-neoplastic cells	50	40	10	0
		% neoplastic cells	60	% neoplastic cells	30	50	15	5
8	3	% non-neoplastic cells	0	% non-neoplastic cells	60	40	0	0
		% neoplastic cells	85	% neoplastic cells	5	35	40	20
9	3	% non-neoplastic cells	0	% non-neoplastic cells	30	55	15	0
		% neoplastic cells	80	% neoplastic cells	50	30	20	0
10	3	% non-neoplastic cells	0	% non-neoplastic cells	25	60	15	0
		% neoplastic cells	90	% neoplastic cells	20	30	40	10
